# Novel and optimized strategies for inducing fibrosis *in vivo*: focus on Duchenne Muscular Dystrophy

**DOI:** 10.1186/2044-5040-4-7

**Published:** 2014-08-25

**Authors:** Patrizia Pessina, Daniel Cabrera, María Gabriela Morales, Cecilia A Riquelme, Jaime Gutiérrez, Antonio L Serrano, Enrique Brandan, Pura Muñoz-Cánoves

**Affiliations:** 1 Cell Biology Group, Department of Experimental and Health Sciences, CIBER on Neurodegenerative Diseases (CIBERNED), Pompeu Fabra University (UPF), Dr. Aiguader, 88, 08003 Barcelona, Spain; 2 Department of Cell and Molecular Biology, Catholic University of Chile, Avenida Libertador Bernardo O’Higgins, 340, Santiago, Chile; 3 Institució Catalana de Recerca i Estudis Avançats (ICREA), Dr. Aiguader, 88, 08003 Barcelona, Spain

## Abstract

**Background:**

Fibrosis, an excessive collagen accumulation, results in scar formation, impairing function of vital organs and tissues. Fibrosis is a hallmark of muscular dystrophies, including the lethal Duchenne muscular dystrophy (DMD), which remains incurable. Substitution of muscle by fibrotic tissue also complicates gene/cell therapies for DMD. Yet, no optimal models to study muscle fibrosis are available. In the widely used mdx mouse model for DMD, extensive fibrosis develops in the diaphragm only at advanced adulthood, and at about two years of age in the ‘easy-to-access’ limb muscles, thus precluding fibrosis research and the testing of novel therapies.

**Methods:**

We developed distinct experimental strategies, ranging from chronic exercise to increasing muscle damage on limb muscles of young mdx mice, by myotoxin injection, surgically induced trauma (laceration or denervation) or intramuscular delivery of profibrotic growth factors (such as TGFβ). We also extended these approaches to muscle of normal non-dystrophic mice.

**Results:**

These strategies resulted in advanced and enhanced muscle fibrosis in young mdx mice, which persisted over time, and correlated with reduced muscle force, thus mimicking the severe DMD phenotype. Furthermore, increased fibrosis was also obtained by combining these procedures in muscles of normal mice, mirroring aberrant repair after severe trauma.

**Conclusions:**

We have developed new and improved experimental strategies to accelerate and enhance muscle fibrosis *in vivo*. These strategies will allow rapidly assessing fibrosis in the easily accessible limb muscles of young mdx mice, without necessarily having to use old animals. The extension of these fibrogenic regimes to the muscle of non-dystrophic wild-type mice will allow fibrosis assessment in a wide array of pre-existing transgenic mouse lines, which in turn will facilitate understanding the mechanisms of fibrogenesis. These strategies should improve our ability to combat fibrosis-driven dystrophy progression and aberrant regeneration.

## Background

In skeletal muscle, accumulation of collagens (fibrosis) in the extracellular matrix (ECM) is most often associated with the muscular dystrophies, characterized by muscle wasting, leading to loss of patient mobility. Duchenne muscular dystrophy (DMD) is one of the severest of the dystrophies and is caused by loss of the dystrophin protein due to genetic mutations. As a result, the sarcolemma becomes fragile and susceptible to contraction-induced damage [1]. Skeletal muscle stem cells (satellite cells) mediate the repair process, but in the absence of dystrophin, the muscle undergoes continuous cycles of degeneration and regeneration, eventually leading to satellite cell depletion and myofiber loss [2-4]. The severity of this childhood-associated pathology may also be exacerbated by the growth of myofibers that occurs in boys with DMD over many years [5]. Affected children eventually succumb to muscle wasting, with muscle progressively being replaced by fat and fibrotic tissue, leading to premature death in the late teens or early twenties from respiratory and cardiac failure [6]. There are currently only palliative treatments for DMD patients. Importantly, no effective clinical treatments are available yet to combat or attenuate fibrosis in patients with DMD. Halting or diminishing the development of fibrosis could not only ameliorate DMD progression, but could also increase the success of new cell- and gene-based therapies [7,8]. The mdx mouse strain, the most widely used animal model for studying human DMD, has a nonsense mutation in dystrophin exon 23 leading to dystrophin protein absence [9]. Although mdx mice and DMD patients share many genetic, biochemical and histological similarities, the clinical manifestations are generally less severe in mdx mice [10]. While DMD individuals have a high degree of muscle fibrosis, mdx mice present extensive fibrosis exclusively in the diaphragm muscle. In the limb muscles of mdx mice, however, fibrosis only becomes apparent around 20 months of age [11]. Therefore, despite our awareness of the importance of fibrosis in DMD, there is a lack of appropriate mouse models for studying dystrophic skeletal muscle fibrosis in accessible muscles, such as limb muscles, without requiring nearly two years for fibrosis to appear. Thus, there is a genuine need to develop mouse models that present fibrosis at early stages in life and that more closely mimic human DMD.

In this manuscript, we present several experimental strategies to simply and effectively advance and enhance muscle fibrosis in young mdx mice. We use both physiological exercise, as well as more direct tissue-damaging procedures or delivery of profibrotic growth factors to limb muscle of young dystrophic mice, and demonstrate sustained collagen deposition reminiscent of aged mdx diaphragm muscle, and muscle of human DMD patients. Notably, we could extend these strategies to induce fibrosis in muscles of normal, non-dystrophic mice, which will facilitate studying fibrosis in a wide array of genetically modified mouse lines, and this will in turn increase our understanding of the cells and molecules involved in fibrosis development. Thus, we offer for the first time, to the best of our knowledge, a comparative and quantitative set of new and improved strategies for inducing muscle tissue fibrosis, which will greatly foster our ability to combat fibrosis-dependent dystrophy progression.

## Methods

### Mice handling and sample collection

All experiments were approved by the Ethics Committee of the Pompeu Fabra University (UPF) and performed according to Spanish and European legislation. Mice were housed in standard cages under 12-hour light–dark cycles and fed *ad libitum* with a standard chow diet. Three-month-old normal C57Bl/6 J mice (the classic standard laboratory mouse strain, hereafter referred to as WT) and dystrophic C57Bl/10scsn-mdx (mdx) male mice were used in experiments: the background strain for mdx mice is similar to, but not identical with, the C57Bl/6 J strain. All operations were performed after injection intraperitoneal (i.p.) of ketamine/metedomidine anesthesia (50 mg/kg and 1 mg/kg body weight). Atipamezol (1.0 mg/kg body weight) by subcutaneous injection was used to reverse the effects of anesthesia. Mice were sacrificed at the indicated ages and the tissues were immediately processed to avoid artifacts, either by direct freezing in liquid nitrogen for protein and RNA extraction or in 2-methylbutane cooled with liquid nitrogen for histological analysis, as described below.

### Skeletal muscle fibrogenic treatments

– **Chronic exercise**: Mdx mice were exercised three times per week on a treadmill for 30 minutes at a speed of 12 meters per minute, with a rest of 5 minutes every 10 minutes of exercise. Mdx mice of three, four and five months of age were exercised for three, two and one month, respectively and were sacrificed at the age of six months, together with age-matched unexercised control mdx mice. At the end of the training period muscles were collected and processed for further analyses.

– **Single treatments in WT and mdx mice**:

• **Myotoxin-induced injuries**:

• *Cardiotoxin injury:* Tibialis anterior (TA) muscles of three-month-old WT or mdx mice were injected with 50 μl of 10^–5^ M cardiotoxin (CTX; Latoxan, Rosans, France). Muscles were collected at the indicated times on each set of experiments, which was usually two weeks after myotoxin injection. Muscle samples were also obtained at one month post-injection (in WT mice) and two months post-injection (in mdx mice).

• *Barium chloride injury:* TA muscle of three-month-old WT mice was injected with 50 μl of 0.2% barium chloride (BaCl_2_) and was isolated after two weeks. For repeated BaCl_2_ injuries, BaCl_2_ injections were made in the same muscle, one per week for six weeks, and muscles were isolated and collected for analysis two weeks after the last injection.

• **Traumatic injuries**:

• *Laceration:* TA muscles of three-month-old WT or mdx mice were subjected to laceration (LAC) as previously described [11,12]. Briefly, the skin was carefully cut and separated from the underlying tissue, then the TA muscle of one leg was cut horizontally at its middle of the length by making a lesion through 75% of their width and 50% of the muscle thickness with a scalpel. Contralateral control muscles were sham-operated. In WT mice, muscles were collected at two weeks and one month post-surgery. In mdx mice, muscles were obtained at two weeks and two months post-surgery.

• *Denervation*: Muscle denervation (DEN) was performed as previously described [13,14]. In brief, a 5 mm segment of the sciatic nerve was surgically removed down to the gluteus maximum from the right legs. In three-month-old WT mice, TA muscles were isolated for analysis at two weeks and one month post-surgery. In three-month-old mdx mice, TA muscles were collected at two weeks and two months post-surgery. Contralateral muscles of sham-operated mice were used as controls on every single treatment (CTX, BaCl_2_, LAC and DEN).

• **Profibrotic growth factor treatments:**

• *Transforming growth factor beta treatment*: 50 ng of transforming growth factor beta 1 (TGFβ1) (recombinant human TGFβ1; R&D Systems, Minneapolis, MN, USA) were injected in the TA muscle in a volume of 50 μl of phosphate-buffered saline (PBS, vehicle). Two injections (one per week) were made in TA muscle of three-month-old mdx mice, and muscles were collected for analysis two weeks or two months after the first injection.

• *Connective tissue growth factor delivery*: TA muscles of three-month-old mdx mice were injected with 1 × 10^11^ viral particles of Ad-m connective tissue growth factor (CTGF) or Ad-GFP or with PBS (vehicle) [15] as a control in a total volume of 50 μl. Muscles were collected two weeks after injury.

– **Combined treatments in WT mice:**

*Cardiotoxin injury combined with denervation:* TA muscles of three-month-old WT mice were injected with 50 μl of 10^–5^ M CTX immediately after DEN, as indicated above. Muscles were collected at two weeks and one month after the treatments. Contralateral muscles of non-denervated left legs were used as controls.

*Cardiotoxin injury combined with TGFβ/CTGF treatment:* TA muscles of three-month-old WT mice were injected with 50 μl of 10^–5^ M CTX. TGFβ1 was injected intramuscularly twice at day 7 and 10 after cardiotoxin injection. Muscles were collected at two weeks and one month after the cardiotoxin injection. Contralateral muscles of sham-operated legs were used as controls. When indicated, CTGF adenoviral delivery was performed immediately after cardiotoxin injection.

Specific information about starting and sampling ages of mice after the different experimental protocols is included in Table S1 in Additional file 1.

### Dystrophic patients study

Human samples were provided from Dr. J. Colomer (Hospital Sant Joan de Deu, Barcelona, Spain). DMD diagnosis was established on a total absence of dystrophin by immunohistochemistry and Western blotting. Muscle samples were obtained by a standard quadriceps muscle biopsy from six DMD patients (ranging from five to eleven years of age) and five healthy male human controls of similar age (seven to fourteen years). Quantification of fibrosis was carried out by color image segmentation and automatic measurement using Fiji image analysis software [16]. The ratio of the total area of fibrosis to the total biopsy area was used to estimate the extent of fibrosis (fibrosis index). Histological analysis was performed similarly to mouse samples, as explained in the next section.

### Histological analysis and immunohistochemistry

Cryosections (10 μm thickness) were stained with hematoxylin/eosin (H&E) or Sirius red (Sigma-Aldrich, St Louis, MO, USA). Quantification of collagen content in muscle was performed according to Ardite *et al*. [11]. Briefly, 10 cryosections were collected in a tube and were sequentially incubated with a solution containing 0.1% Fast green in saturated picric acid, washed with distillated water, incubated with 0.1% Fast green and 0.1% Sirius red in saturated picric acid, washed with distillated water, and gently resuspended in a solution of 0.1 M NaOH in absolute methanol (1 vol:1 vol). Absorbance was measured in a spectrophotometer at 540 and 605 nm wavelengths and used to calculate total protein and collagen.

Immunohistochemistry on frozen sections was performed using the following primary antibodies: rabbit polyclonal collagen I (Coll 1) (Millipore, Billerica, MA, USA), rabbit polyclonal fibronectin (FN) (Abcam, Cambridge, MA, USA) and rabbit polyclonal phosphorylated-Smad2/3 (P-Smad2/3) (Abcam). For immunoperoxidase staining, labeling of sections was performed using the peroxidase staining kit (Vector Laboratories, Burlingame, CA, USA) according to the manufacturer’s instructions. For immunofluorescence, secondary antibodies were coupled to Alexa Fluor 488 or 568 fluorochromes (Invitrogen, Carlsbad, CA, USA). Stained sections were photographed on a Leica DM6000B microscope (Leica Microsystems, Wetzler, Germany).

### RNA isolation, reverse transcription (RT) and real-time quantitative PCR

Total RNA was isolated from muscle tissue using Trizol (Invitrogen). cDNA was synthesized from 1 μg of total RNA using the First Strand cDNA Synthesis kit and random priming according to the manufacturer’s instructions (Promega, Madison, WI, USA). RT-PCR was performed on a LightCycler 480 System using LightCycler 480 SYBR Green I Master Mix (Roche, Basel, Switzerland) with 10 μM each primer and normalized to L7 ribosomal RNA as a housekeeping gene: mL7 5′-GAAGCTCATCTATGAGAAGGC–3′ and 5′–AAGACGAAGGAGCTGCAGAAC-3′; mCollagen I, 5′-GGTATGCTTGATCTGTATCTGC-3′ and 5′-AGTCCAGTTCTTCATTGCATT-3′; mCTGF, 5′-CAGGCTGGAGAAGCAGAGTCGT-3′ and 5′-CTGGTGCAGCCAGAAAGCTCAA–3′; mTIMP-1 5′-TTCCAGTAAGGCCTGTAGC-3′ and 5′-TTATGACCAGGTCCGAGTT-3′; mTGFβ 5′-TATGACCAGGTCCGAGTT-3′ and 5′-CTGGTGCAGCCAGAAAGCTCAA-3′; hFibronectin: 5′- GGATGACAAGGAAAATAGCCCTG-3′ and 5′-GAACATCGGTCACTTGCATCT-3′; hTIMP-1 5′-CTTCTGCAATTCCGACCTCGT-3′ and 5′-CCCTAAGGCTTGGAACCCTTT-3′; hTGFβ 5′-CCTAA GGCCAGATCCTGTCCAAGC-3′ and 5′- GTGGGTTTCCACCATTAGCAC-3′; hCTGF 5′- CAAGGGCCTCTTCTGTGACT-3′ and 5′-ACGTGCACTGGTACTTGCAG-3′.

### Quantification of TGFβ protein

The protein concentration of active and total (active plus latent) TGFβ1 levels in dystrophic muscle was quantified by ELISA (Promega), following the manufacturer’s instructions.

### Muscle force measurement

Muscle strength was determined as described previously [17]. Briefly, after the indicated days of treatment, mice were sacrificed and the TA was rapidly excised into a dish containing oxygenated Krebs-Ringer solution. The optimum muscle length (Lo) was determined from micromanipulations of muscle length to produce the maximum isometric twitch force. Maximum isometric-specific tetanic force was determined from the plateau of the curve of the relationship between specific isometric force with a stimulation frequency (Hz) ranging from 1 to 200 Hz for 450 ms, with 2 minutes of rest between stimuli. The force was normalized per total muscle fiber cross-sectional area (CSA), to calculate the specific net force (mN/mm^2^).

### Statistical analysis

Comparison between groups was done using the nonparametric Mann–Whitney *U* test for independent samples, with a confidence level of 95% being considered statistically significant. One-way or two-way analysis of variance (ANOVA) was used for comparisons between multiple groups as appropriate, and *post hoc* analysis was performed using Tukey’s test. All statistical analyses were performed using GraphPad Prism 5.0 (GraphPad Software, San Diego, CA, USA). The number of samples analyzed per group is detailed on each figure. Differences were considered to be statistically significant at *P* <0.05.

## Results

### Mdx mice reproduce the human DMD fibrotic phenotype in aging diaphragm muscle

**Figure 1:**
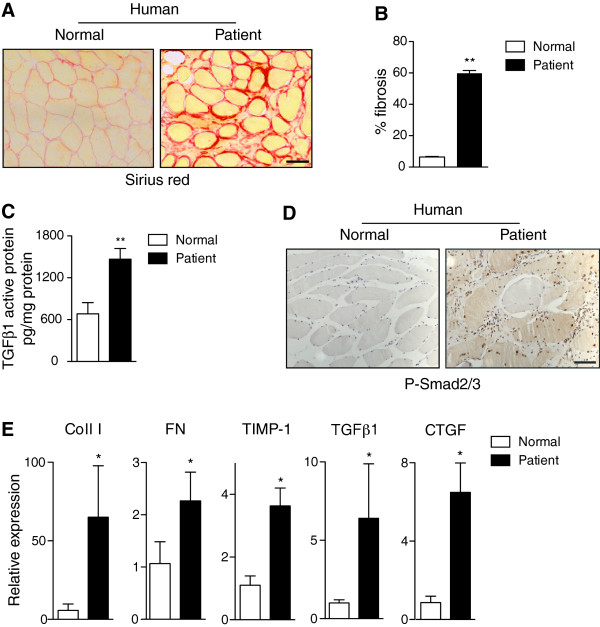
**Quantification of fibrosis in human dystrophic muscle. (A)** Representative Sirius red staining of healthy and dystrophic human muscle sections reveals the extent of collagen deposition in patients with Duchenne muscular dystrophy (DMD). **(B)** Percentage of fibrosis (collagen content) in healthy and DMD muscles as measured by Sirius red staining in muscle sections. Data correspond to the mean ± SEM; n = 6 for DMD group and n = 5 for control group. Non-parametric Mann–Whitney *U* test was used for comparison. **P <0.01 versus healthy controls. **(C)** Active transforming growth factor beta 1 (TGFβ1) protein levels measured by ELISA in muscle biopsy material from healthy and DMD muscle. Data correspond to the mean ± SEM; n = 5 on each group. Non-parametric Mann–Whitney *U* test; **P <0.01 versus healthy controls. **(D)** Immunohistochemistry for phosphorylated-Smad2/3 (P-Smad2/3) in healthy and dystrophic human muscle sections. **(E)** Quantitative RT-PCR for collagen I (Coll 1), fibronectin (FN), tissue inhibitor of metalloproteinases 1(TIMP-1), TGFβ1 and connective tissue growth factor (CTGF) in DMD muscles compared to healthy muscles (which were given the arbitrary value of 1). Data correspond to the mean ± SEM; n = 4 on each group. Non-parametric Mann–Whitney *U* test *P <0.05. Scale bars = 50 μm.

To recreate as closely as possible the fibrosis status of human DMD in animal models, we first sought to characterize in detail distinct fibrosis-associated parameters in muscle biopsies of DMD patients. Compared to muscles of healthy individuals, we found an increased collagen content in DMD patients, based on Sirius red staining and collagen quantification, where fibrotic tissue had replaced the myofiber area (Figure 1A and B). Transforming growth factor-β (TGFβ) has been shown to be a profibrotic cytokine in many types of fibrotic tissues and is a potent stimulator of matrix production, including collagen, by fibroblasts [13,18-22]. We found higher levels of activated TGFβ protein in muscle biopsies from dystrophic children compared to healthy controls (Figure 1C). Consistent with this, we found enhanced levels of active Smad2/3 (as indicated by phosphorylated Smad2/3) (Figure 1D) and TGFβ target genes such as Coll I, FN, tissue inhibitor of metalloproteinases 1 (TIMP-1) and CTGF, indicative of functional TGFβ signaling in fibrotic DMD muscle (Figure 1E).

**Figure 2:**
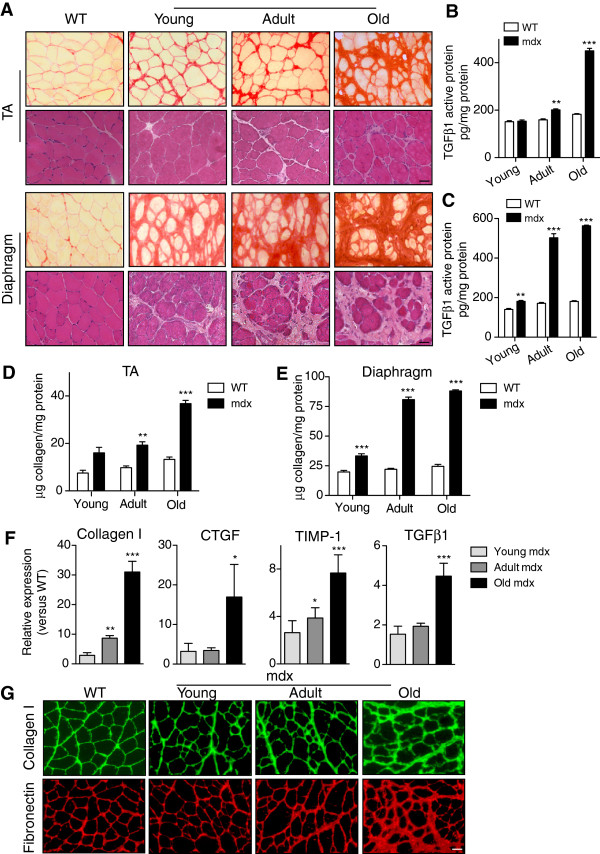
**Quantification of fibrosis in muscle of mdx mice. (A)** Sirius red and H&E staining of mdx tibialis anterior (TA) (upper panels) and diaphragm (lower panels) muscles at different ages compared to adult wild-type (WT) muscle. ‘Young’ corresponds to muscles of three-month-old mice, ‘Adult’ to nine months and ‘Old’ to eighteen to twenty-four months of age. **(B)** and **(C)** Active transforming growth factor beta 1 (TGFβ1) protein quantification by ELISA in TA and diaphragm muscles of WT and mdx mice at the indicated ages, respectively. Data correspond to the mean ± SEM values; n = 4 for each group. Two-way analysis of variance with Tukey’s *post hoc* multiple comparison test. **P <0.01, ***P <0.001 versus age-matched WT. **(D)** and **(E)** Quantification of collagen content in TA and diaphragm muscles of WT and mdx mice at different ages. Values are mean ± SEM; n = 4 for each group. Two-way analysis of variance with Tukey’s *post hoc* multiple comparison test. **P <0.01, ***P <0.001 versus control WT values. **(F)** Relative expression of collagen I, connective tissue growth factor (CTGF), tissue inhibitor of metalloproteinases 1(TIMP-1) and TGFβ1 mRNA by quantitative RT-PCR in mdx TA muscles at the indicated ages with respect to WT muscles (baseline set arbitrarily to 1). Values are mean ± SEM; n = 3 for each group. Two-way analysis of variance with Tukey’s *post hoc* multiple comparison test. *P <0.05, **P <0.01, ***P <0.001, versus age-matched WT. **(G)** Representative pictures of immunofluorescence staining for collagen I (green) and fibronectin (red) in young, adult and old mdx TA, compared to WT muscle. Scale bars = 50 μm.

The most common experimental model of DMD is the mdx mouse [23]. We examined the TA limb muscle and the diaphragm muscle by hematoxylin and eosin (H&E) and Sirius red staining from young (three months of age), adult (nine months) and old mdx mice (eighteen to twenty-four months) in comparison to age-matched WT muscles. Significant fibrosis, similar to that observed in human patients, was found in TA muscles of mdx mice only at old age (>18 months) (Figure 2A, upper panels and Figure 2D), while adult mdx TA muscles presented milder fibrosis. In diaphragm muscle, fibrosis increased age-dependently, reaching near maximum levels in adult mice of nine months of age and plateauing thereafter (Figure 2A, lower panels). Furthermore, in TA muscles of mdx mice, collagen content, activated TGFβ and expression of ECM-associated molecules started to increase at adult age but were much higher at old age (Figure 2B, D, F, G); in the diaphragm muscle, these parameters were moderately increased already at young age (Figure 2C and E, and Figure S1A and B in Additional file 2). The limited development of fibrosis (compared to the diaphragm muscle) in the easily accessible limb muscles of mdx mice until old age, reinforces the need for developing new protocols that will advance muscle fibrosis in young mdx mice.

### Exercise training triggers fibrosis in muscles of young dystrophic mice

**Figure 3:**
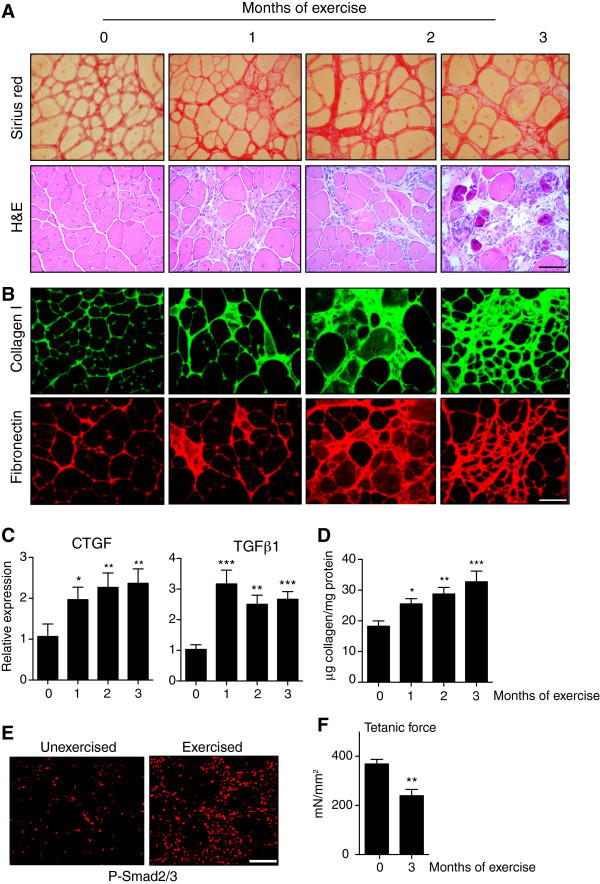
**Effect of exercise on muscle fibrosis in mdx mice. (A)** Sirius Red and H&E staining of gastrocnemius muscle of mdx mice that were exercised three times weekly, for 30 minutes at a speed of 12 meters per minute with a rest of 5 minutes each 10 minutes of exercise, for one, two and three months, compared to sections of muscle from unexercised mdx mice. All the samples were collected when the animals were six months old (see the Methods section). **(B)** Representative immunofluorescence for collagen I and fibronectin in muscle sections of control or exercised mice as shown in **(A)**. **(C)** Quantitative RT-PCR of connective tissue growth factor (CTGF) and transforming growth factor beta 1 (TGFβ1) mRNA levels after exercising for the indicated period as compared to unexercised age-matched mdx mice. Data correspond to the mean ± SEM; n = 4 sedentary and 4 exercised mdx mice for each exercise time point. One-way analysis of variance with Tukey’s *post hoc* multiple comparison test; *P <0.05, **P <0.01, ***P <0.001 versus control. **(D)** Biochemical quantification of collagen protein content in mdx gastrocnemius muscle after exercising for the indicated periods, as compared to unexercised age-matched mdx mice. Data correspond to the mean ± SEM; n = 4 sedentary and 4 exercised mdx mice for each exercise time point. One-way analysis of variance with Tukey’s *post hoc* multiple comparison test; *P <0.05, **P <0.01, ***P <0.001 versus control. **(E)** Immunofluorescence for phosphorylated-Smad2/3 proteins on sections from gastrocnemius muscle of six-month-old mdx mice after three months of exercise, as evidence for TGFβ activation, compared to unexercised age-matched control mdx mice. **(F)***Ex vivo* maximum isometric force (tetanic force) of gastrocnemius muscle of age-matched unexercised and three-month-trained mdx mice. Values as mean ± SEM; n = 7 on each group. Non-parametric Mann–Whitney *U* test; **P <0.01 versus non-exercised. Scale bars = 50 μm.

In a first attempt to induce and advance muscle fibrosis, young mdx mice were subjected to a chronic exercise training routine, known to exacerbate the muscle degeneration/regeneration process [24]. Three-month-old mdx mice were exercised on a treadmill three times per week for up to three months, for 30 minutes each time, at a speed of 12 meters per minute, with a rest of 5 minutes every 10 minutes [25]. Muscles of mice exercised for one, two and three months were compared with age- and sex-matched unexercised mice. After one month, exercised mdx mice already showed a worsening of the dystrophic phenotype (compared to age-matched controls), and this condition was further aggravated by continued adherence to the exercise regime. Hindlimb muscles (gastrocnemius and TA) of one-month exercised mice displayed a higher degree of fibrosis, identified by Sirius red staining, with respect to normally active non-exercised mdx control mice (Figure 3A, and Figure S2A in Additional file 3). The increased fibrosis observed by Sirius red staining was confirmed by Coll I immunofluorescence (Figure 3B, upper panels, and Figure S2B in Additional file 3), and the greater deposition of FN (Figure 3B, and Figure S2B in Additional file 3, lower panels) that is normally only observed in old mdx limb muscles (Figure 2G). Consistent with this, collagen content and the expression of TGFβ1 and CTGF mRNA, and the levels of P-Smad2/3 proteins, were increased in exercised dystrophic mdx muscles, compared to non-exercised controls (Figure 3C, D, E, and Figure S2C in Additional file 3). Furthermore maximum force of the muscles of mdx mice subjected to the exercise regime was decreased with respect to non-exercised mice (Figure 3F, and Figure S2D in Additional file 3). These data confirm that exercise in young mdx mice can activate fibrogenesis, and in particular the profibrotic TGFβ pathway, and thereby enhance and anticipate muscle tissue fibrosis.

### Surgical muscle injuries advance and enhance fibrosis in young dystrophic mice

**Figure 4:**
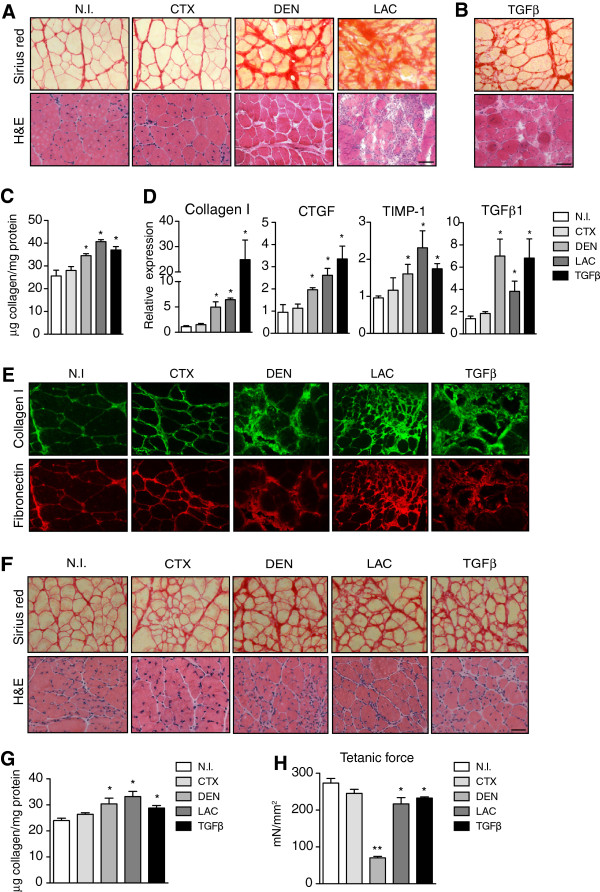
**Induction of fibrosis after chemical and surgical muscle damage in young mdx mice. (A)** Sirius red and hematoxylin and eosin (H&E) staining of tibialis anterior (TA) muscles of young (three-month-old) mdx mice two weeks after cardiotoxin (CTX)-injury (50 μl of 10^–5^ M), denervation (DEN) and laceration (LAC), compared to non-injured (NI) muscle of sham-operated mdx mice. **(B)** Sirius red and H&E staining of young mdx TA muscle analyzed after two sequential treatments with recombinant transforming growth factor beta 1 (TGFβ1) (50 ng in 50 μl phosphate-buffered saline (PBS)) spaced seven days apart. **(C)** Biochemical quantification of collagen protein content in mdx TA, two weeks after different treatments relative to uninjured mdx control. Values represent mean ± SEM; n = 4 on each group. Non-parametric Mann–Whitney *U* test; *P <0.05 versus NI. **(D)** Quantitative RT-PCR of collagen I, connective tissue growth factor (CTGF), tissue inhibitor of metalloproteinases 1(TIMP-1) and TGFβ1 mRNA expression in mdx muscle two weeks after different injuries versus control mdx mice. Values represent mean ± SEM; n = 4 on each group. Non-parametric Mann–Whitney *U* test; *P <0.05 versus NI. **(E)** Representative immunostaining for collagen I (green) and fibronectin (red) on sections of young mdx TA muscles two weeks after injury relative to control. **(F)** Sirius Red and H&E staining of mdx TA muscle two months after CTX injury, DEN, LAC or injection of TGFβ in three-month-old mdx muscle compared to NI mdx control muscle. **(G)** Quantification of collagen content in TA muscle of young mdx mice two months after different treatments, as described above. Values represent mean ± SEM; n = 4 on each group. Non-parametric Mann–Whitney *U* test; *P <0.05 versus NI. **(H)***Ex vivo* maximum isometric force (tetanic force) of TA muscle of young mdx mice two months after treatments. Values as mean ± SEM; n = 4 to 5 on each group. Non-parametric Mann–Whitney *U* test; *P <0.05; **P <0.01 versus NI. Scale bars = 50 μm.

We next sought alternative and faster ways than long-term exercise training to induce fibrosis in limb muscles of young mdx mice, based on inflicting increased surgical or chemical damage. Since CTX-induced muscle injury is a widely used and well-characterized experimental model for inducing skeletal muscle degeneration/regeneration [26-28], we hypothesized that superimposing CTX-induced damage on young dystrophic mdx muscle would promote fibrosis. Despite an early increase in collagen content, two weeks after intramuscular CTX injection (50 μl of 10^–5^ M), TA mdx muscle showed a similar quantity of deposited collagen compared to non-injured (NI) mdx TA indicating that the fibrogenic effect of CTX-induced damage was transient (Figure 4A). To increase and prolong collagen deposition, we superimposed on young mdx limb muscle two more extreme, but distinct, experimental paradigms: laceration (LAC) and denervation (DEN). The DEN model involves severing the sciatic nerve thus causing atrophy of the denervated myofibers [14,29], while the LAC model consists in a deep cut across the muscle, which causes a delay in the healing process [11,12]. Muscle of dystrophic mdx mice at two weeks after DEN showed an increased deposition of collagen relative to CTX-injured mdx muscle (Figure 4A, C). Lacerated dystrophic muscle also showed increased fibrosis after two weeks, which was even higher than in denervated muscle after the same time period (Figure 4A, C). Importantly, the mdx muscle fibrosis induced by both methods persisted for up to two months, as indicated by histological and biochemical parameters (see below, Figure 4F and G). This extended fibrotic status reinforces the utility of these two methods as drivers of limb muscle fibrosis in young mdx mice, after which the tissue more closely resembles the more severe phenotype of old mdx mice, as well as human DMD patients. Furthermore, these procedures have the advantage of not requiring exercise devices, nor the time and labor of the three-month exercise protocol.

### Raising TGFβ levels in dystrophic muscle of young mdx mice accelerates fibrosis and accentuates disease severity

Despite the profibrotic effect of the surgical methods on mdx muscle, each one has particularities and limitations. In the LAC model, the injury is confined to a small area of the muscle and this reduces the amount of tissue available for further studies, whereas, for reasons of animal welfare, DEN can only be performed in one leg of the mouse, affecting only the muscles under the knee. Therefore, based on our observation of the elevated levels of TGFβ in human and mouse dystrophic muscle (Figures 1 and 2), and its correlation with the extent of dystrophy-associated fibrosis, we reasoned that exogenous delivery of TGFβ1 to muscle of young dystrophic mice might increase and accelerate the development of fibrosis. We therefore performed two intramuscular TA injections of TGFβ1 (50 ng of TGFβ1 in 50 μl of PBS per injection), spaced seven days apart, in an attempt to sustain the profibrogenic action of this growth factor. Contralateral control muscles received the same number of injections of PBS. Analysis of the muscles histologically by H&E and Sirius red staining showed that TGFβ1 delivery lead to substantial increase in collagen deposition already at two weeks after the first injection, which persisted for up to two months and this was also confirmed by biochemical quantification of muscle extracts (Figure 4B and C). Of note, local muscle overexpression of the TGFβ1 target gene product CTGF also increased fibrogenesis in limb muscle of young mdx mice (Figure S3A, B in Additional file 4).Overall, comparing the distinct biochemical and functional parameters in all the procedures tested revealed that LAC and TGFβ treatments gave statistically higher quantitative measures of collagen than NI age-matched control mdx muscles. The collagen values for LAC and TGFβ1 treatments were comparable to the values recorded in limb muscles of old mdx mice (see Figure 2D), indicating that either one of these methods advances fibrosis by the equivalent of about fourteen months (that is inducing fibrosis at four months of age instead of eighteen months). DEN also significantly increases muscle collagen content over mdx controls, but to a lesser extent than LAC or TGFβ1 treatment (Figure 4A and C). Interestingly, the levels of endogenous TGFβ1 mRNA were increased in young dystrophic muscle in response to LAC, DEN and exogenous TGFβ1 delivery, but not CTX. Consistent with this, the expression of TGFβ-dependent signaling fibrotic target genes, such as, Coll I, CTGF, TIMP-1, were increased in mdx limb muscle after all three treatments, but not in CTX-damaged muscle (Figure 4D). Finally, immunostaining for FN and Coll I on sections from the different damaged mdx muscles showed greater ECM production than uninjured (or CTX-injured) dystrophic muscle (Figure 4E).Remarkably, at two months after injury, collagen deposition still persisted in TGFβ-treated young dystrophic muscles as it did in lacerated and denervated muscles, as revealed by histological and biochemical analysis (Figure 4F and G). In agreement with this, and demonstrating the deleterious physiological consequences of the increased fibrosis in young mdx muscles, the maximum force of the muscles subjected to the distinct treatments decreased with respect to NI mdx muscles (Figure 4H), therefore better mimicking the severe phenotype of the human condition.

### Induction of fibrosis in non-dystrophic, wild-type muscle by combining surgical injury and growth factor delivery

Fibrosis persistence has negative consequences on tissue wound healing. Severe muscle injuries caused by trauma often result in scar formation at the expense of tissue repair. Thus, we designed easy-to-perform profibrotic procedures in non-dystrophic WT muscle, which could ideally be extended to a wide variety of transgenic mouse lines for research or therapeutic purposes.

**Figure 5:**
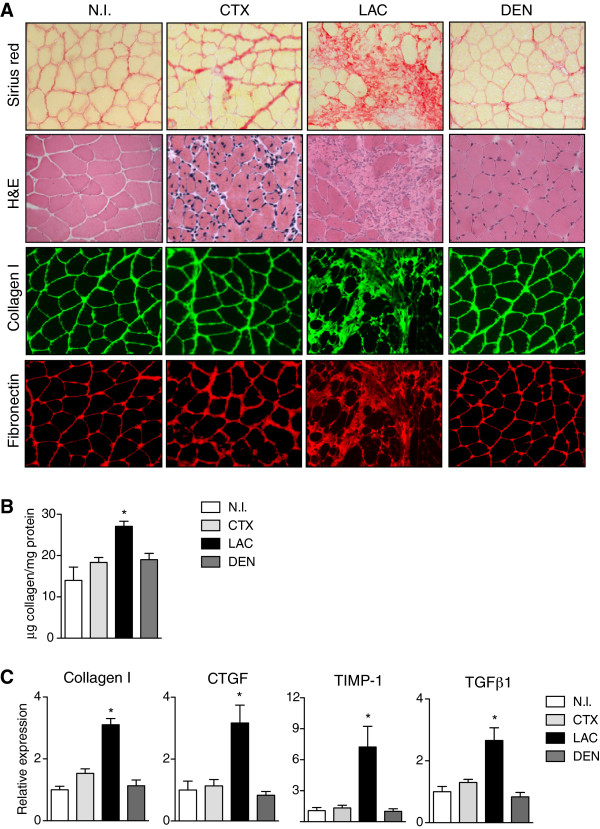
**Quantification of muscle fibrosis after chemical and surgical damage in wild-type mice. (A)** Sirius red, hematoxylin and eosin (H&E), collagen I (green) and fibronectin (red) staining on wild-type (WT) tibialis anterior (TA) muscles two weeks after cardiotoxin (CTX) injury (50 μl of 10^–5^ M), laceration (LAC) and denervation (DEN) compared to non-injured (NI) muscle of sham-operated WT mice. **(B)** Quantification of collagen content in WT muscle after different injuries. Data correspond to the mean ± SEM; n = 4 on each group. Non-parametric Mann–Whitney *U* test; *P <0.05 versus NI. **(C)** Quantitative RT-PCR for collagen I, connective tissue growth factor (CTGF), tissue inhibitor of metalloproteinases 1 (TIMP-1) and transforming growth factor beta 1 (TGFβ1) mRNA in muscles after the different injuries (values are means ± SEM; n = 4 on each group. Non-parametric Mann–Whitney *U* test; *P <0.05 versus NI). Scale bar = 50 μm.

**Figure 6:**
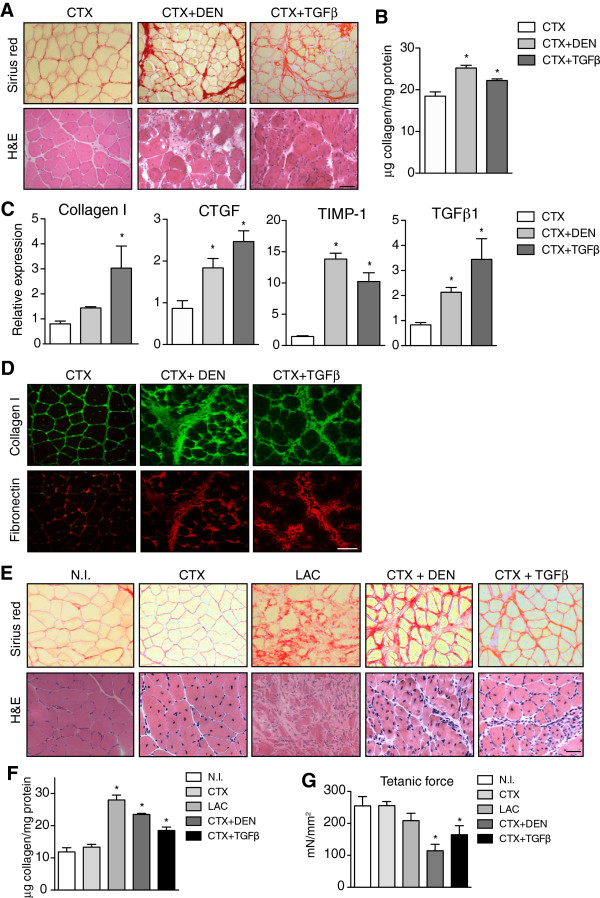
**Synergistic effect on fibrosis induction in muscle of wild-type mice by combined treatments. (A)** Sirius red and hematoxylin and eosin (H&E) staining of wild-type (WT) tibialis anterior (TA) muscles subjected to a combination of cardiotoxin (CTX) injury (50 μl of 10^–5^ M) and denervation or transforming growth factor beta 1 (TGFβ1) (50 ng in 50 μl phosphate-buffered saline (PBS)) injection (as described in the Methods section), respectively, compared to CTX injury alone. **(B)** Quantification of collagen content in muscle after each treatment. Data correspond to the mean ± SEM; n = 4 on each group. Non-parametric Mann–Whitney *U* test; *P <0.05 versus CTX injury. **(C)** Quantitative RT-PCR for collagen I, connective tissue growth factor (CTGF), tissue inhibitor of metalloproteinases 1 (TIMP-1) and TGFβ1 after the different treatments (n = 4 on each group. Non-parametric Mann–Whitney *U* test; *P <0.05, compared to CTX injury). **(D)** Representative immunostaining for collagen I (green) and fibronectin (red) on sections of WT muscle subjected to the different fibrosis-inducing methods. **(E-G)** Analysis of long-term fibrosis in WT muscle at one month after injury. Data are compared to non-injured (NI) muscle of sham-operated WT mice. **(E)** Sirius red and H&E staining of CTX-injured, lacerated, CTX/denervation and CTX/TGFβ-injured muscles at one month after injury. **(F)** Quantification of collagen content one month after injury of WT muscle. Values represent mean ± SEM; n = 4 on each group. Non-parametric Mann–Whitney *U* test; *P <0.05 versus NI. **(G)***Ex vivo* maximum isometric force (tetanic force) of TA muscle. Values as mean ± SEM; n = 4 on each group. Non-parametric Mann–Whitney *U* test; *P <0.05 versus NI. Scale bars = 50 μm.

We applied the surgical/chemical methods previously used on muscle of dystrophic mdx mice (see above), either alone or in combination, to induce muscle fibrosis in WT mice. First, we performed CTX injury in TA muscle of WT mice and assessed fibrosis development. We observed a mild and transient deposition of ECM between days 5 and 7 after CTX (50 μl of 10^–5^ M) muscle injury (Figure S4A and B in Additional file 5); however, it did not persist beyond this stage. Indeed, two weeks after CTX injury, collagen content returned to near basal levels, in agreement with efficient muscle recovery (Figure 5A-C and Figure 6H).We next subjected WT muscle to the more severe LAC and DEN procedures and compared the fibrosis index of the affected muscles to that of CTX-injured muscle at similar time points. LAC in TA muscle of WT mice disrupted the tissue quite extensively and for a prolonged period of time (over one month) resulting in sustained fibrosis, which correlated with the slow kinetics for regeneration (Figure 5A, B). DEN, in turn, did not alter ECM production significantly, as revealed by H&E and Sirius red staining, or immunostaining for Coll I and FN, despite inducing the expected myofiber atrophy. Consistent with these findings, we only observed statistically significant increases in the expression of TGFβ1 and the fibrotic markers Coll I, FN, CTGF and TIMP-1 in lacerated muscle, but not in denervated or CTX-injured muscles, compared to uninjured muscle (Figure 5C). These results suggest that LAC is the most fibrotic of the traumatic models tested in non-dystrophic mice.

As stated above, one of the limitations of the LAC procedure is the restricted availability of biopsy material. Trying to induce fibrosis by methods that would render more fibrotic tissue available for analysis, we decided to combine CTX injury, which individually was a poor fibrosis-inducing method, with either DEN or co-injection of TGFβ1 in muscle of WT mice, methods which we previously showed were able to increase fibrosis in young mdx muscle (Figure 4). Both DEN and injection of TGFβ1 failed to induce fibrosis in WT muscles when used alone (Figure 5, and Figure S4C and D in Additional file 5). Accordingly, TA muscles of WT mice were first subjected to CTX injection (50 μl of 10^–5^ M) and subsequently denervated or injected twice with TGFβ1 (50 ng of TGFβ1 in 50 μl PBS per injection) (at day 7 and 10 after CTX injection) and muscles were collected two and four weeks later. We found that the combination of CTX injury with DEN or TGFβ1 delivery induced fibrosis significantly compared to CTX injury alone, as shown by Sirius red staining (Figure 6A), collagen quantification as well as expression of fibrotic markers by quantitative RT-PCR and immunohistochemistry analyses, after 14 days (Figure 6B-D), correlating with delayed regeneration kinetics (Figure 6A). Of note, a combination of CTX injury and CTGF local overexpression produced similar profibrotic effects as combining CTX injury and TGFβ1 delivery (Figure S3C in Additional file 4), suggesting that part of the TGFβ profibrotic actions are likely to be mediated by CTGF.We next compared the persistence of fibrosis over time and the consequences on muscle function of each of the distinct fibrogenic regimes on WT muscle. Sirius red staining and collagen quantification showed that muscle fibrosis still persisted after four weeks of either laceration or CTX combined with TGFβ1 or DEN, compared to muscle injured with CTX alone or NI muscle (Figure 6E and 6F). The relevance of these results was supported by functional studies of WT muscle after the combined profibrotic treatments (CTX combined with TGFβ1 or DEN). Indeed, dual treatments on muscles exerted a synergistic effect, resulting in increased fibrosis and reduced net force compared to uninjured muscle or muscle injured with CTX alone (Figure 6G). These results suggest that in WT mice, LAC, as well as a combination of CTX injury with either DEN or TGFβ1, proved to be effective fibrosis-inducing models that trigger a rapid accumulation of fibrotic tissue that is sustained for an extended period of time, with negative consequences on muscle function.

**Figure 7:**
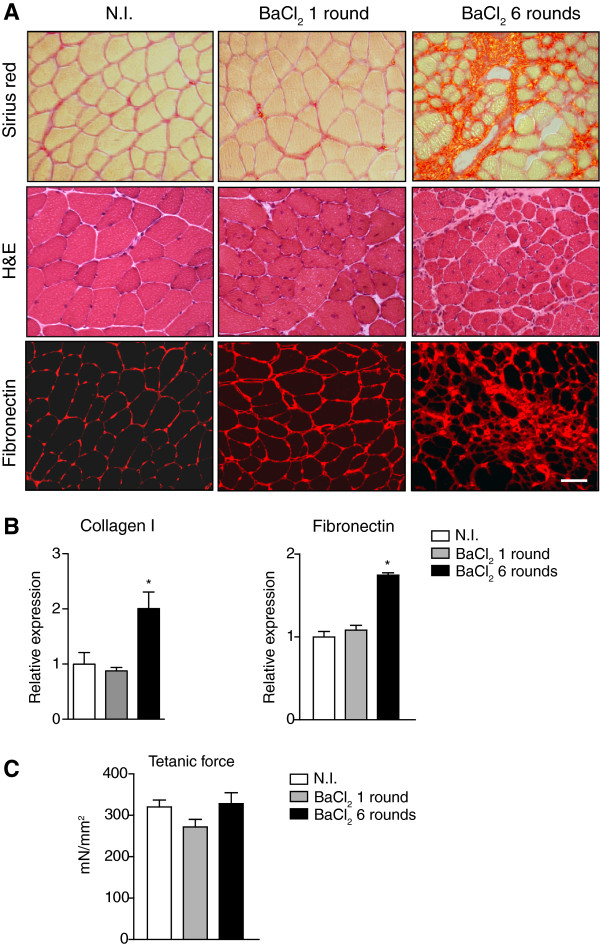
**Fibrosis induction in muscle of wild-type mice after repeated BaCl**_**2 **_**injuries. (A)** Sirius red, H&E and fibronectin staining on wild-type (WT) tibialis anterior (TA) muscles subjected to one or six consecutive weekly rounds of BaCl_2_ injections (50 μl of 0.2% BaCl_2_), compared to non-injured (NI) muscle of sham-operated WT mice, sampled two weeks after the final injection. **(B)** Quantitative expression of fibronectin and collagen I in the distinct muscle samples (values are means ± SEM; n = 4 to 5 on each group. Non-parametric Mann–Whitney *U* test; **P <0.05 versus NI). **(C)***Ex vivo* maximum isometric force (tetanic force) of TA muscle. Values as mean ± SEM; n = 4 for each group; non-parametric Mann–Whitney *U* test; no significant differences *P* >0.05. Scale bar = 50 μm.

Finally, and in order to further expand the variety of fibrogenic-inducing procedures to the maximum number of laboratories working on skeletal muscle, we tested the fibrosis-inducing effect of a widely used muscle-damaging method involving BaCl_2_ injection in WT muscle. We found that, as for CTX injection, one intramuscular injection of BaCl_2_ (50 μl of 0.2% BaCl_2_) only induced a very mild and transient accumulation of ECM. Of note, repeated injections (spaced one week) for up to six weeks resulted in significant ECM accumulation after eight weeks from the first injection (that is two weeks after the last injection), although no major change in muscle force was observed (Figure 7A-C). Thus, repeated damaging with myotoxins may be a fibrosis-inducing alternative in non-dystrophic muscle, although development of fibrosis requires up to eight weeks, and involves weekly mouse manipulation for six weeks, compared to the less labor-consuming and more rapid fibrogenic effect (with additional impact on muscle force) of the combined treatments.

## Discussion

Muscular dystrophies constitute a heterogeneous group of inherited myopathies, characterized by progressive muscular degeneration, of which DMD is one of the severest. Progressive replacement of skeletal muscle by fat and fibrotic tissue not only exacerbates disease progression, but also impairs the efficiency of gene- and stem cell-based therapies [30,31]. Yet, there is no effective clinical treatment to reverse or attenuate fibrosis in DMD patients, except for promising new agents such as halofuginone [32]. To a great extent, this deficit may derive from the poor understanding of the mechanisms underlying fibrogenesis in muscular dystrophy. Indeed, chronic inflammation and production of collagens by myoblasts are among the few reported causal factors promoting progression to fibrosis in dystrophic muscle [33-38]. The largely unknown etiology of fibrogenesis in DMD in turn may be principally due to the lack of adequate animal models of muscle fibrosis. Here we report the application of simple models of tissue damage that are able to significantly enhance the fibrotic response in skeletal muscle and which may be useful for investigating therapeutic strategies for DMD.

Studies using mdx mice, the most common mouse model of DMD, may not be translated directly to dystrophic patients due to the mild phenotype they display. In particular, limb muscles of mdx mice show a relatively efficient regeneration and no significantly aberrant deposition of ECM proteins until very old age. Progressive endomysial fibrosis only develops in diaphragm muscle, but is still not significantly advanced until well into adulthood [39]. To try to accelerate or exacerbate this phenotype, other mouse models have been generated such as mdx mice lacking arginase-2, PAI-1 (plasminogen activator inhibitor-1) or Cmah (cytidine monophosphate-sialic acid hydroxylase) [11,40,41] and previously the mdx/utrn^+/-^ mouse line (mdx mice with haploinsufficiency of utrophin) [42]. However, mdx/utrn^+/-^ mice show early mortality and the manipulation of the line requires time and resources in genotyping and breeding. Moreover, the genetics of these mouse models do not adequately reflect human DMD patients. Therefore, the need for fibrotic models that do not require waiting for the natural physiological onset of fibrosis in the hindlimb of old mice, and that recapitulate the human DMD phenotype becomes increasingly more important. One recent attempt to address this problem came from Desguerre and colleagues (2012) who described a model of mechanical muscle injury by daily repeated micro-punctures in mdx hindlimb muscle [43]. Induction of endomysial fibrosis in dystrophic muscle through this method is ascribed to a small fibrotic area and requires daily animal manipulation during two weeks. In addition, this procedure does not seem to induce fibrosis in WT mice [43].

The strategies we propose here are valid alternatives to both hasten the appearance and prolong the duration of fibrosis in hindlimb muscles of young mdx mice, with very limited (non-daily) animal manipulation, which notably are also able to induce relatively sustained fibrosis in WT muscle. Therefore, these methods would be applicable to other genetically modified mice, and this will help further delineating the cellular and genetic basis of muscle fibrosis. Exercise training of young mdx mice induced endomysial fibrosis, resembling the phenotype of old hindlimb dystrophic muscles; however, although this method can be considered more physiological, it still requires a lengthy time period to obtain a fibrotic muscle tissue, in addition to significant amount of effort and time, since exercise protocols need to be applied several times a week for ideally three months. At variance, the methods based on muscle growth factor delivery and surgical injuries that we present here offer a faster and less labor-intensive alternative. The rationale for the proposed profibrotic growth factor-based methods relies on the observation that, in fibrotic muscles of human DMD patients and old mdx mice, TGFβ1 (and its downstream target CTGF) is present at high levels [22,44], correlating with the increased activation of Smad2/3 transcriptional mediators (see Figure S5 in Additional file 6). Of the surgical methods tested, muscle laceration proved to be the most effective for inducing sustained fibrosis; however, this method has the disadvantage that the affected area is relatively small (and only one muscle per mouse can be lesioned due to the severity of the procedure) thereby limiting the amount of material available for downstream processing. Subsequent cellular analysis of fibrotic muscle by techniques such as fluorescence-activated cell sorting (FACS) may not be possible in this type of model without vast improvements of sensitivity or without increasing the number of animals used, which has extra cost and ethical implications. Sciatic nerve denervation of mdx mice generates increased collagen deposition, as a possible mechanism to replace the tissue volume lost due to myofiber atrophy. All of these fibrogenesis-inducing methods persist with time, since at two months after injury muscles still displays a fibrotic phenotype. Moreover, consistent with the idea that fibrosis aggravates muscle dysfunction in DMD, we showed that maximal muscle force was also reduced in young mdx mice after fibrosis induction through the different protocols.

Finally, to be able to investigate fibrosis development and therapeutic options in muscle of non-dystrophic models, we sought to apply these methods to WT mice. To date, studies on muscle damage in non-dystrophic models have been performed classically with a single injection of myotoxins (for example, CTX or BaCl_2_). Despite the general use, we have shown in this study that these standard single-injury methods are not appropriate fibrosis-inducing models, as the resolution of the damage occurs rapidly and collagen deposition is very mild and only transient. On the contrary, muscle laceration of WT muscle induces a massive collagen deposition that is relatively stable over long periods of time, despite affecting only a localized small tissue area. However, the combination of regimes showed an improved capacity to generate fibrosis in WT muscle for a sustained period of time, correlating with reduction in muscle force, indicating that they mimic in WT animals pathophysiological situations of severe muscle trauma that result in aberrant regeneration, scar deposition and functional impairment. We propose that this variety of fibrosis-inducing methodologies will enable fibrosis to be studied in a vast array of transgenic mouse lines (with no apparent underlying muscle pathology) or after crossing them with dystrophic strains such as mdx mice.

## Conclusions

Collectively, through this study, we propose novel and/or optimized experimental strategies to accelerate, anticipate and boost muscle fibrosis in young dystrophic mice or to drive *de novo* fibrosis onset in WT mice. We think that our findings provide very useful methodologies that will facilitate research in the emerging field of skeletal muscle fibrosis. In particular, these rapid and feasible procedures for most laboratories will help getting deeper insight into the mechanisms underlying muscle fibrosis, as well as developing therapeutic strategies aimed to reduce its magnitude in dystrophic diseases and to ameliorate dystrophy progression. Since fibrosis is also a main obstacle for stem cell engraftment, availability of appropriate fibrosis models will be a determinant factor in the research toward successful gene/cell therapy-based strategies in muscular dystrophy.

## Abbreviations

BaCl_2_: barium chloride; Coll I: collagen I; CTGF: connective tissue growth factor; CTX: cardiotoxin; DEN: denervation; DMD: Duchenne muscular dystrophy; ECM: extracellular matrix; FN: fibronectin; H&E: hematoxylin and eosin; LAC: laceration; NI: non-injured; PBS: phosphate-buffered saline; P-Smad2/3: phosphorylated Smad2/3; TA: tibialis anterior; TGFβ1: transforming growth factor beta1; TIMP-1: tissue inhibitor of metalloproteinases 1; WT: wild-type.

## Competing interests

The authors declare that they have no competing interests.

## Authors’ contributions

PMC, EB and ALS conceived and designed the project. PP performed and analyzed most of the experiments and was assisted by DC, MGM, CAR and JG for Figures 3 and 7. PMC and PP wrote the manuscript and ALS and EB revised and edited it. All authors read and approved the final manuscript.

## Supplementary Material

Additional file 1: Table S1 Methods and sampling times of the different fibrosis-inducing procedures in mdx and wild-type (WT) mice. Click here for file

Additional file 2: Figure S1 Quantification of fibrosis in mdx diaphragm muscle. **(A)** Relative expression of collagen I, connective tissue growth factor (CTGF), tissue inhibitor of metalloproteinases 1(TIMP-1) and transforming growth factor beta 1 (TGFβ1) mRNA by quantitative RT-PCR in mdx diaphragm muscles at the indicated ages respect to wild-type (WT) muscles. Values are mean ± SEM; n = 4 for each group; non-parametric Mann–Whitney *U* test; *P <0.05 versus age-matched WT. **(B)** Representative pictures of immunofluorescence staining for collagen I (green) and fibronectin (red) in young, adult and old mdx diaphragm, compared to age-matched WT muscle. Scale bars = 50 μm. Click here for file

Additional file 3: Figure S2 Effect of exercise on tibialis anterior (TA) mdx muscle. **(A)** Sirius red and hematoxylin and eosin (H&E) staining of TA muscle of mdx mice that were exercised three times weekly, for 30 minutes at a speed of 12 meters per minute with a rest of 5 minutes each 10 minutes of exercise, for one, two and three months, compared to sections of muscle from unexercised mdx mice. All the samples were collected when the animals were six months old (see the Methods section). **(B)** Representative immunofluorescence for collagen I and fibronectin in muscle sections of control and exercised mice as shown in (A). **(C)** Biochemical quantification of collagen protein content in mdx TA muscle after exercising for the indicated period as compared to unexercised age-matched mdx mice. Data correspond to the mean ± SEM; n = 4 sedentary and 4 exercised mdx mice for each exercise time point. One-way analysis of variance with Tukey’s *post hoc* multiple comparison test; **P <0.01, ***P <0.001 versus control. **(D)***Ex vivo* maximum isometric force (tetanic force) of TA muscle of age-matched unexercised and three-month-trained mdx mice. Values as mean ± SEM; n = 7 on each group. Non-parametric Mann–Whitney *U* test; **P <0.01 versus non-exercised. Scale bars = 50 μm. Click here for file

Additional file 4: Figure S3 Fibrosis induction in muscle by viral delivery of connective tissue growth factor (CTGF). **(A)** Mdx mice: Sirius red and hematoxylin and eosin (H&E) staining of mdx tibialis anterior (TA) muscles overexpressing mouse CTGF after intramuscular injection of 50 μl of 2x10^11^ particles of adenovirus (AdV) in three-month-old mice. **(B)** Collagen content quantification. Data correspond to the mean ± SEM; n = 4 on each group. Non-parametric Mann–Whitney *U* test; *P <0.05 versus NI. **(C)** Wild-type (WT) mice: H&E of WT muscles after adenoviral CTGF delivery coupled with cardiotoxin (CTX) injury; representative immunostaining for collagen I (green) and fibronectin (red) on sections of AdV-transduced muscle overexpressing CTGF. Scale bars = 50 μm. Click here for file

Additional file 5: Figure S4 Collagen deposition after cardiotoxin (CTX)-induced muscle injury and transforming growth factor beta 1 (TGFβ1) delivery alone is quickly resolved in wild-type (WT) mice. **(A)** Sirius red, hematoxylin and eosin (H&E), collagen I (green) and fibronectin (red) staining on WT tibialis anterior (TA) muscles after five and eight days from CTX injury, compared to non-injured (NI) muscle of sham-operated WT mice. **(B)** Quantification of collagen content in muscle after treatment. Data correspond to the mean ± SEM, n = 4 on each group. Non-parametric Mann–Whitney *U* test; *P <0.05 versus NI. **(C)** Sirius red, H&E, collagen I (green) and fibronectin (red) staining on WT TA muscle two weeks after two sequential treatments with recombinant TGFβ1 (50 ng in 50 μl phosphate-buffered saline (PBS)), spaced seven days apart. **(D)** Quantification of collagen content in muscle after injection of TGFβ1 or PBS (vehicle). Data are mean ± SEM, n = 4 for each group. Non-parametric Mann–Whitney *U* test; no significant differences *P* >0.05. Scale bars = 50 μm. Click here for file

Additional file 6: Figure S5 Smad2/3 protein phosphorylation in injured muscles. Immunofluorescence for phosphorylated-Smad2/3 proteins on sections from tibialis anterior (TA) muscle of mdx **(A)** and wild-type (WT) **(B)** mice after the indicated treatments. Scale bars = 50 μm. Click here for file

## References

[B1] Emery AE The muscular dystrophies Lancet 2002 359 687 695 11879882 10.1016/S0140-6736(02)07815-7

[B2] Briggs D Morgan JE Recent progress in satellite cell/myoblast engraftment - relevance for therapy FEBS J 2013 280 4281 4293 23560812 10.1111/febs.12273 PMC3795440

[B3] Serrano AL Munoz-Canoves P Regulation and dysregulation of fibrosis in skeletal muscle Exp Cell Res 2010 316 3050 3058 20570674 10.1016/j.yexcr.2010.05.035

[B4] Yablonka-Reuveni Z Anderson JE Satellite cells from dystrophic (mdx) mice display accelerated differentiation in primary cultures and in isolated myofibers Dev Dynamics 2006 235 203 212 10.1002/dvdy.20602 16258933

[B5] Grounds MD Shavlakadze T Growing muscle has different sarcolemmal properties from adult muscle: a proposal with scientific and clinical implications: reasons to reassess skeletal muscle molecular dynamics, cellular responses and suitability of experimental models of muscle disorders Bio Essays 2011 33 458 468 10.1002/bies.201000136 21500235

[B6] Muntoni F Cardiac complications of childhood myopathies J Child Neurol 2003 18 191 202 12731645 10.1177/08830738030180030301

[B7] Benedetti S Hoshiya H Tedesco FS Repair or replace? Exploiting novel gene and cell therapy strategies for muscular dystrophies FEBS J 2013 280 4263 4280 23387802 10.1111/febs.12178

[B8] Tedesco FS Hoshiya H D’Antona G Gerli MF Messina G Antonini S Tonlorenzi R Benedetti S Berghella L Torrente Y Kazuki Y Bottinelli R Oshimura M Cossu G Stem cell-mediated transfer of a human artificial chromosome ameliorates muscular dystrophy Sci Trans Med 2011 3 96ra78 10.1126/scitranslmed.3002342 21849666

[B9] Sicinski P Geng Y Ryder-Cook AS Barnard EA Darlison MG Barnard PJ The molecular basis of muscular dystrophy in the mdx mouse: a point mutation Science 1989 244 1578 1580 2662404 10.1126/science.2662404

[B10] Carnwath JW Shotton DM Muscular dystrophy in the mdx mouse: histopathology of the soleus and extensor digitorum longus muscles J Neuro Sci 1987 80 39 54 10.1016/0022-510x(87)90219-x 3612180

[B11] Ardite E Perdiguero E Vidal B Gutarra S Serrano AL Munoz-Canoves P PAI-1-regulated miR-21 defines a novel age-associated fibrogenic pathway in muscular dystrophy J Cell Biol 2012 196 163 175 22213800 10.1083/jcb.201105013 PMC3255978

[B12] Menetrey J Kasemkijwattana C Fu FH Moreland MS Huard J Suturing versus immobilization of a muscle laceration. A morphological and functional study in a mouse model Am J Sports Med 1999 27 222 229 10102105 10.1177/03635465990270021801

[B13] Serrano AL Murgia M Pallafacchina G Calabria E Coniglio P Lomo T Schiaffino S Calcineurin controls nerve activity-dependent specification of slow skeletal muscle fibers but not muscle growth Proc Natl Acad Sci USA 2001 98 13108 13113 11606756 10.1073/pnas.231148598 PMC60832

[B14] Glass DJ Skeletal muscle hypertrophy and atrophy signaling pathways Int J Biochem Cell Biol 2005 37 1974 1984 16087388 10.1016/j.biocel.2005.04.018

[B15] Morales MG Cabello-Verrugio C Santander C Cabrera D Goldschmeding R Brandan E CTGF/CCN-2 over-expression can directly induce features of skeletal muscle dystrophy J Pathol 2011 225 490 501 21826667 10.1002/path.2952

[B16] Schindelin J Arganda-Carreras I Frise E Kaynig V Longair M Pietzsch T Preibisch S Rueden C Saalfeld S Schmid B Tinevez JY White DJ Hartenstein V Eliceiri K Tomancak P Cardona A Fiji: an open-source platform for biological-image analysis Nat Methods 2012 9 676 682 22743772 10.1038/nmeth.2019 PMC3855844

[B17] Cabello-Verrugio C Morales MG Cabrera D Vio CP Brandan E Angiotensin II receptor type 1 blockade decreases CTGF/CCN2-mediated damage and fibrosis in normal and dystrophic skeletal muscles J Cellular Mol Med 2012 16 752 764 21645240 10.1111/j.1582-4934.2011.01354.x PMC3822846

[B18] Biernacka A Frangogiannis NG Aging and cardiac fibrosis Aging Dis 2011 2 158 173 21837283 PMC3153299

[B19] Brandan E Gutierrez J Role of proteoglycans in the regulation of the skeletal muscle fibrotic response FEBS J 2013 280 4109 4117 23560928 10.1111/febs.12278

[B20] MacDonald EM Cohn RD TGFbeta signaling: its role in fibrosis formation and myopathies Cur Opinion Rheumatol 2012 24 628 634 10.1097/BOR.0b013e328358df34 22918531

[B21] Mann CJ Perdiguero E Kharraz Y Aguilar S Pessina P Serrano AL Munoz-Canoves P Aberrant repair and fibrosis development in skeletal muscle Skelet Muscle 2011 1 21 21798099 10.1186/2044-5040-1-21 PMC3156644

[B22] Morales MG Cabrera D Cespedes C Vio CP Vazquez Y Brandan E Cabello-Verrugio C Inhibition of the angiotensin-converting enzyme decreases skeletal muscle fibrosis in dystrophic mice by a diminution in the expression and activity of connective tissue growth factor (CTGF/CCN-2) Cell Tissue Res 2013 353 173 187 23673415 10.1007/s00441-013-1642-6

[B23] Dangain J Vrbova G Muscle development in mdx mutant mice Muscle Nerve 1984 7 700 704 6543918 10.1002/mus.880070903

[B24] De Luca A Pierno S Liantonio A Cetrone M Camerino C Fraysse B Mirabella M Servidei S Ruegg UT Conte Camerino D Enhanced dystrophic progression in mdx mice by exercise and beneficial effects of taurine and insulin-like growth factor-1 J Pharm Exp Thera 2003 304 453 463 10.1124/jpet.102.041343 12490622

[B25] Morales MG Gutierrez J Cabello-Verrugio C Cabrera D Lipson KE Goldschmeding R Brandan E Reducing CTGF/CCN2 slows down mdx muscle dystrophy and improves cell therapy Hum Mol Gen 2013 22 4938 4951 23904456 10.1093/hmg/ddt352

[B26] Perdiguero E Sousa-Victor P Ruiz-Bonilla V Jardi M Caelles C Serrano AL Munoz-Canoves P p38/MKP-1-regulated AKT coordinates macrophage transitions and resolution of inflammation during tissue repair J Cell Biol 2011 195 307 322 21987635 10.1083/jcb.201104053 PMC3198158

[B27] Suelves M Lopez-Alemany R Lluis F Aniorte G Serrano E Parra M Carmeliet P Munoz-Canoves P Plasmin activity is required for myogenesis in vitro and skeletal muscle regeneration in vivo Blood 2002 99 2835 2844 11929773 10.1182/blood.v99.8.2835

[B28] Suelves M Vidal B Serrano AL Tjwa M Roma J Lopez-Alemany R Luttun A de Lagran MM Diaz-Ramos A Jardi M Roig M Dierssen M Dewerchin M Carmeliet P Muñoz-Cánoves P uPA deficiency exacerbates muscular dystrophy in MDX mice J Cell Biol 2007 178 1039 1051 17785520 10.1083/jcb.200705127 PMC2064626

[B29] Carlson BM Billington L Faulkner J Studies on the regenerative recovery of long-term denervated muscle in rats Rest Neurol Neurosci 1996 10 77 84 10.3233/RNN-1996-10203 21551856

[B30] Gargioli C Coletta M De Grandis F Cannata SM Cossu G PlGF-MMP-9-expressing cells restore microcirculation and efficacy of cell therapy in aged dystrophic muscle Nat Med 2008 14 973 978 18660817 10.1038/nm.1852

[B31] Muir LA Chamberlain JS Emerging strategies for cell and gene therapy of the muscular dystrophies Expert Rev Mol Med 2009 11 e18 19555515 10.1017/S1462399409001100 PMC4890545

[B32] Turgeman T Hagai Y Huebner K Jassal DS Anderson JE Genin O Nagler A Halevy O Pines M Prevention of muscle fibrosis and improvement in muscle performance in the mdx mouse by halofuginone Neuro Dis 2008 18 857 868 10.1016/j.nmd.2008.06.386 18672370

[B33] Alexakis C Partridge T Bou-Gharios G Implication of the satellite cell in dystrophic muscle fibrosis: a self-perpetuating mechanism of collagen overproduction Am J Physiol Cell Physiol 2007 293 C661 C669 17475662 10.1152/ajpcell.00061.2007

[B34] Morrison J Palmer DB Cobbold S Partridge T Bou-Gharios G Effects of T-lymphocyte depletion on muscle fibrosis in the mdx mouse Am J Pathol 2005 166 1701 1710 15920155 10.1016/S0002-9440(10)62480-7 PMC1602408

[B35] Vidal B Serrano AL Tjwa M Suelves M Ardite E De Mori R Baeza-Raja B Martinez de Lagran M Lafuste P Ruiz-Bonilla V Jardí M Gherardi R Christov C Dierssen M Carmeliet P Degen JL Dewerchin M Muñoz-Cánoves P Fibrinogen drives dystrophic muscle fibrosis via a TGFbeta/alternative macrophage activation pathway Genes Dev 2008 22 1747 1752 18593877 10.1101/gad.465908 PMC2492661

[B36] Vidal B Ardite E Suelves M Ruiz-Bonilla V Janue A Flick MJ Degen JL Serrano AL Munoz-Canoves P Amelioration of Duchenne muscular dystrophy in mdx mice by elimination of matrix-associated fibrin-driven inflammation coupled to the alphaMbeta2 leukocyte integrin receptor Hum Mol Gen 2012 21 1989 2004 22381526 10.1093/hmg/dds012 PMC3315206

[B37] Villalta SA Nguyen HX Deng B Gotoh T Tidball JG Shifts in macrophage phenotypes and macrophage competition for arginine metabolism affect the severity of muscle pathology in muscular dystrophy Hum Mol Gen 2009 18 482 496 18996917 10.1093/hmg/ddn376 PMC2638796

[B38] Kharraz Y Guerra J Mann CJ Serrano AL Munoz-Canoves P Macrophage plasticity and the role of inflammation in skeletal muscle repair Mediators Inflam 2013 2013 491497 10.1155/2013/491497 PMC3572642 23509419

[B39] Stedman HH Sweeney HL Shrager JB Maguire HC Panettieri RA Petrof B Narusawa M Leferovich JM Sladky JT Kelly AM The mdx mouse diaphragm reproduces the degenerative changes of Duchenne muscular dystrophy Nature 1991 352 536 539 1865908 10.1038/352536a0

[B40] Chandrasekharan K Yoon JH Xu Y deVries S Camboni M Janssen PM Varki A Martin PT A human-specific deletion in mouse Cmah increases disease severity in the mdx model of Duchenne muscular dystrophy Sci Trans Med 2010 2 42ra54 10.1126/scitranslmed.3000692 PMC2950110 20668298

[B41] Wehling-Henricks M Jordan MC Gotoh T Grody WW Roos KP Tidball JG Arginine metabolism by macrophages promotes cardiac and muscle fibrosis in mdx muscular dystrophy PloS one 2010 5 e10763 20505827 10.1371/journal.pone.0010763 PMC2874011

[B42] Zhou L Rafael-Fortney JA Huang P Zhao XS Cheng G Zhou X Kaminski HJ Liu L Ransohoff RM Haploinsufficiency of utrophin gene worsens skeletal muscle inflammation and fibrosis in mdx mice J Neurol Sci 2008 264 106 111 17889902 10.1016/j.jns.2007.08.029 PMC2696235

[B43] Desguerre I Arnold L Vignaud A Cuvellier S Yacoub-Youssef H Gherardi RK Chelly J Chretien F Mounier R Ferry A Chazaud B A new model of experimental fibrosis in hindlimb skeletal muscle of adult mdx mouse mimicking muscular dystrophy Muscle Nerve 2012 45 803 814 22581532 10.1002/mus.23341

[B44] Bernasconi P Di Blasi C Mora M Morandi L Galbiati S Confalonieri P Cornelio F Mantegazza R Transforming growth factor-beta1 and fibrosis in congenital muscular dystrophies Neuromusc Dis 1999 9 28 33 10063832 10.1016/s0960-8966(98)00093-5

