# Supplementary material for: Novel and optimized strategies for inducing fibrosis in vivo: focus on Duchenne Muscular Dystrophy
Source: Skelet Muscle. 2014 Aug 25;4:7. doi: 10.1186/2044-5040-4-7 (PMC4142391; doi:10.1186/2044-5040-4-7)
Supplement: Additional file 1: Table S1 — Methods and sampling times of the different fibrosis-inducing procedures in mdx and wild-type (WT) mice. [file 2044-5040-4-7-S1.pdf]

Supplementary Table 1

Method	Genotype	Starting age (weeks)	Sampling age (weeks)
Chronic exercise (1, 2 and 3 months)	mdx	20, 16 and 12	24 (in all groups)
Cardiotoxin injury (CTX)	mdx	12	14 and 20
Laceration (LAC)	mdx	12	14 and 20
Denervation (DEN)	mdx	12	14 and 20
TGFβ treatment	mdx	12	14 and 20
CTGF treatment	mdx	12	14 and 20
Laceration (LAC)	WT	12	14 and 16
Denervation (DEN)	WT	12	14 and 16
CTX+ DEN	WT	12	14 and 16
CTX + TGFβ/CTGF	WT	12	14 and 16
$BaCl_2$ 6 rounds	WT	12	14 and 19
