# Supplementary figures and images for: Novel and optimized strategies for inducing fibrosis in vivo: focus on Duchenne Muscular Dystrophy
Source: Skelet Muscle. 2014 Aug 25;4:7. doi: 10.1186/2044-5040-4-7 (PMC4142391; doi:10.1186/2044-5040-4-7)

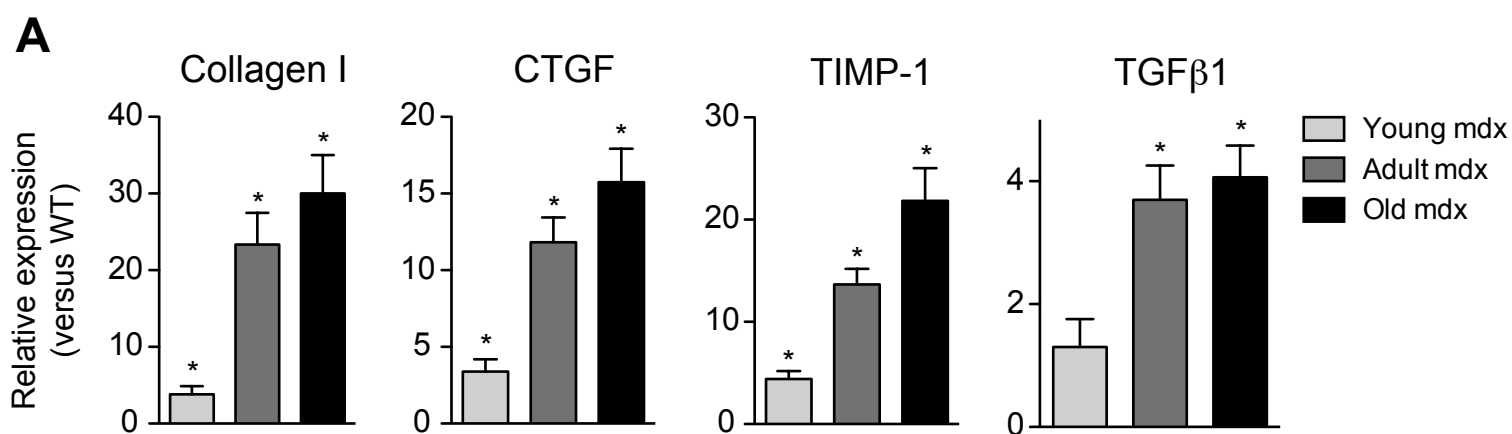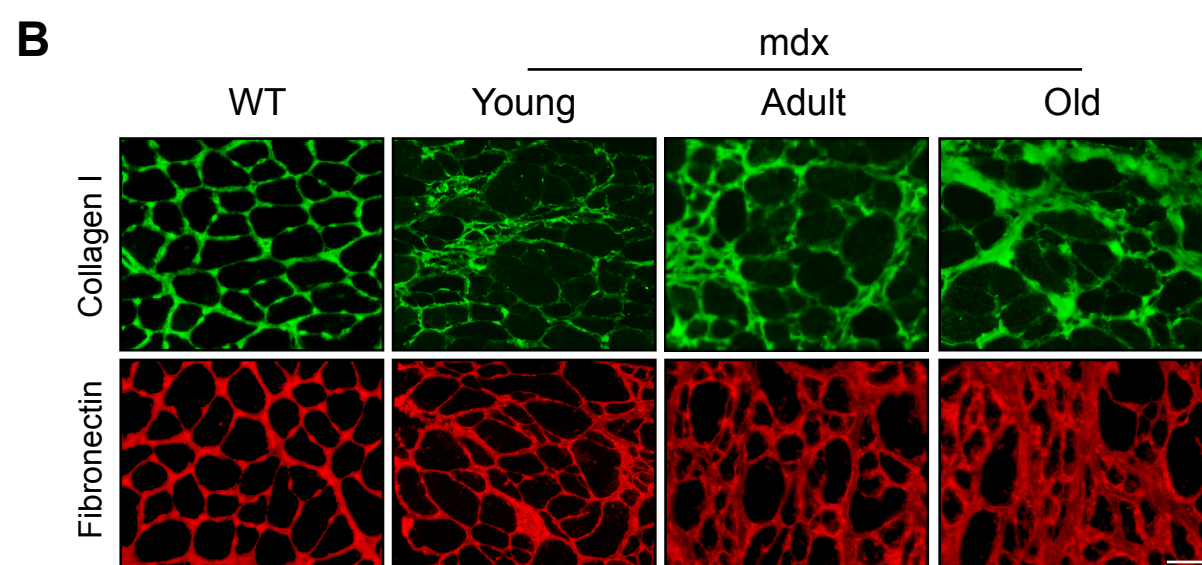

Supplement: Additional file 2: Figure S1 — Quantification of fibrosis in mdx diaphragm muscle. (A) Relative expression of collagen I, connective tissue growth factor (CTGF), tissue inhibitor of metalloproteinases 1(TIMP-1) and transforming growth factor beta 1 (TGFβ1) mRNA by quantitative RT-PCR in mdx diaphragm muscles at the indicated ages respect to wild-type (WT) muscles. Values are mean ± SEM; n = 4 for each group; non-parametric Mann–Whitney U test; *P <0.05 versus age-matched WT. (B) Representative pictures of immunofluorescence staining for collagen I (green) and fibronectin (red) in young, adult and old mdx diaphragm, compared to age-matched WT muscle. Scale bars = 50 μm. [file 2044-5040-4-7-S2.pdf]

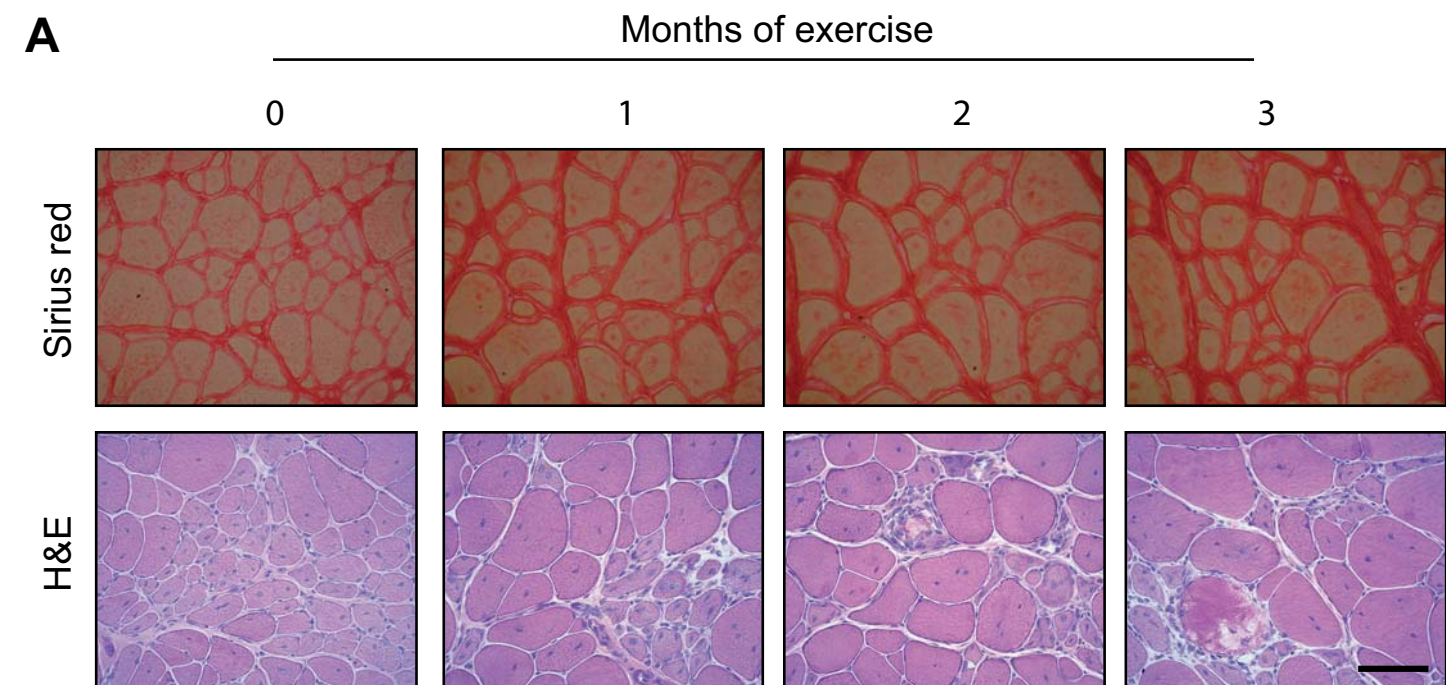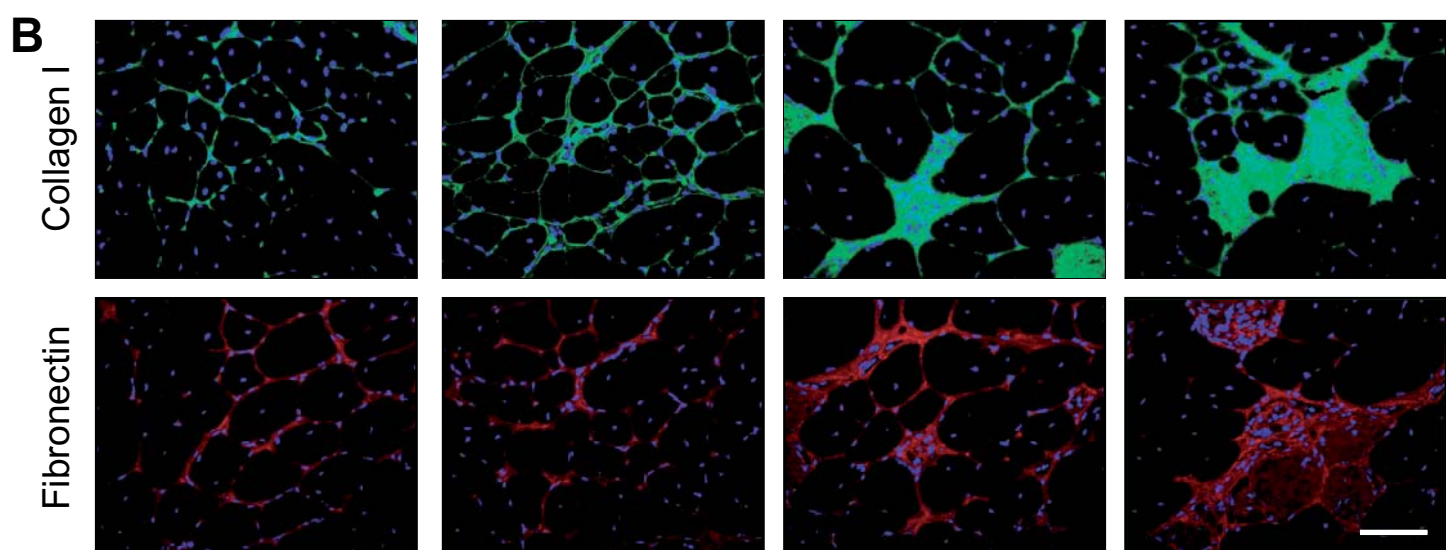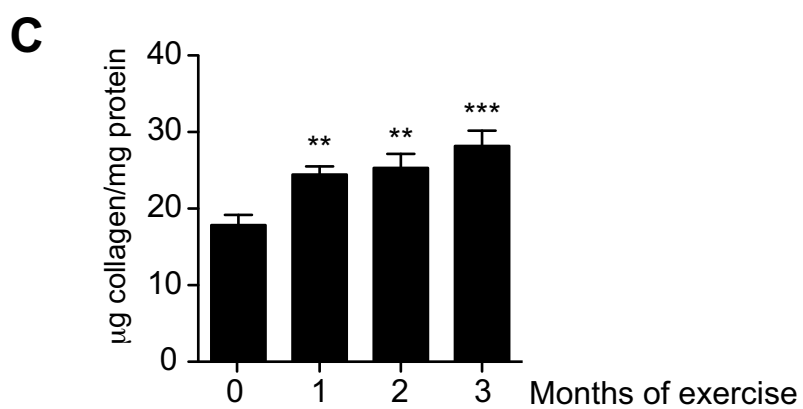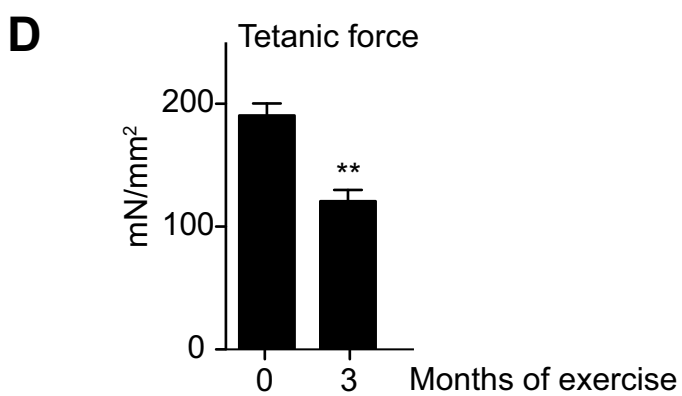

Supplement: Additional file 3: Figure S2 — Effect of exercise on tibialis anterior (TA) mdx muscle. (A) Sirius red and hematoxylin and eosin (H&E) staining of TA muscle of mdx mice that were exercised three times weekly, for 30 minutes at a speed of 12 meters per minute with a rest of 5 minutes each 10 minutes of exercise, for one, two and three months, compared to sections of muscle from unexercised mdx mice. All the samples were collected when the animals were six months old (see the Methods section). (B) Representative immunofluorescence for collagen I and fibronectin in muscle sections of control and exercised mice as shown in (A). (C) Biochemical quantification of collagen protein content in mdx TA muscle after exercising for the indicated period as compared to unexercised age-matched mdx mice. Data correspond to the mean ± SEM; n = 4 sedentary and 4 exercised mdx mice for each exercise time point. One-way analysis of variance with Tukey’s post hoc multiple comparison test; **P <0.01, ***P <0.001 versus control. (D)Ex vivo maximum isometric force (tetanic force) of TA muscle of age-matched unexercised and three-month-trained mdx mice. Values as mean ± SEM; n = 7 on each group. Non-parametric Mann–Whitney U test; **P <0.01 versus non-exercised. Scale bars = 50 μm. [file 2044-5040-4-7-S3.pdf]

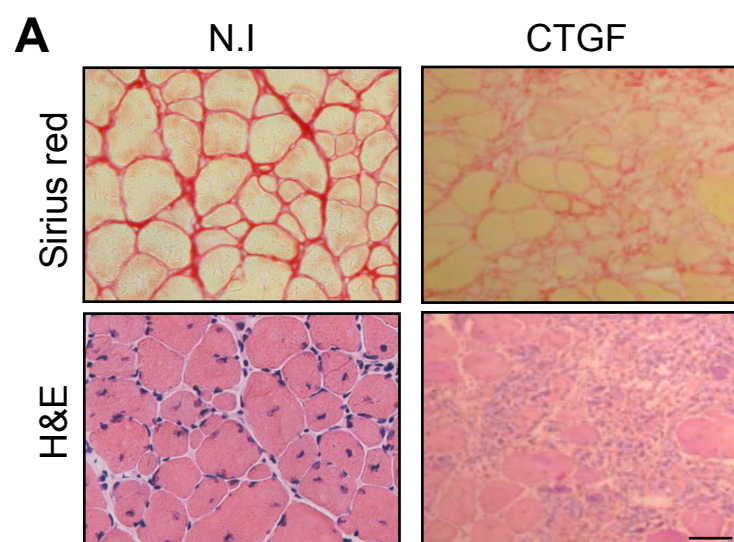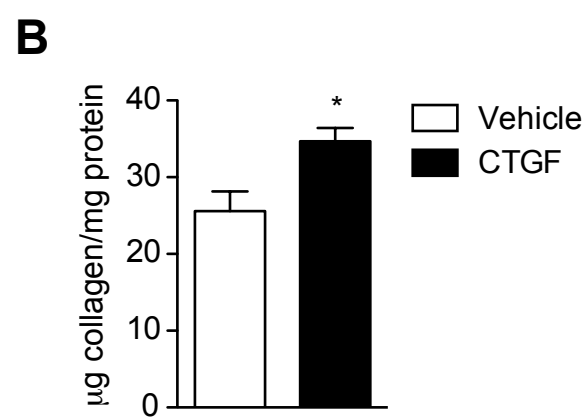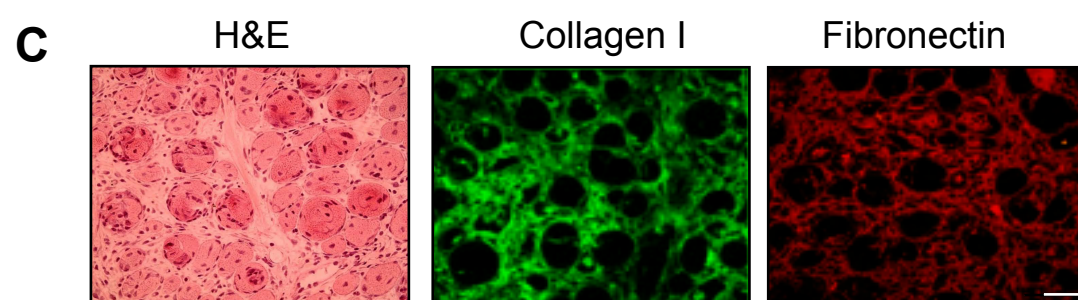

Supplement: Additional file 4: Figure S3 — Fibrosis induction in muscle by viral delivery of connective tissue growth factor (CTGF). (A) Mdx mice: Sirius red and hematoxylin and eosin (H&E) staining of mdx tibialis anterior (TA) muscles overexpressing mouse CTGF after intramuscular injection of 50 μl of 2x10^11^ particles of adenovirus (AdV) in three-month-old mice. (B) Collagen content quantification. Data correspond to the mean ± SEM; n = 4 on each group. Non-parametric Mann–Whitney U test; *P <0.05 versus NI. (C) Wild-type (WT) mice: H&E of WT muscles after adenoviral CTGF delivery coupled with cardiotoxin (CTX) injury; representative immunostaining for collagen I (green) and fibronectin (red) on sections of AdV-transduced muscle overexpressing CTGF. Scale bars = 50 μm. [file 2044-5040-4-7-S4.pdf]

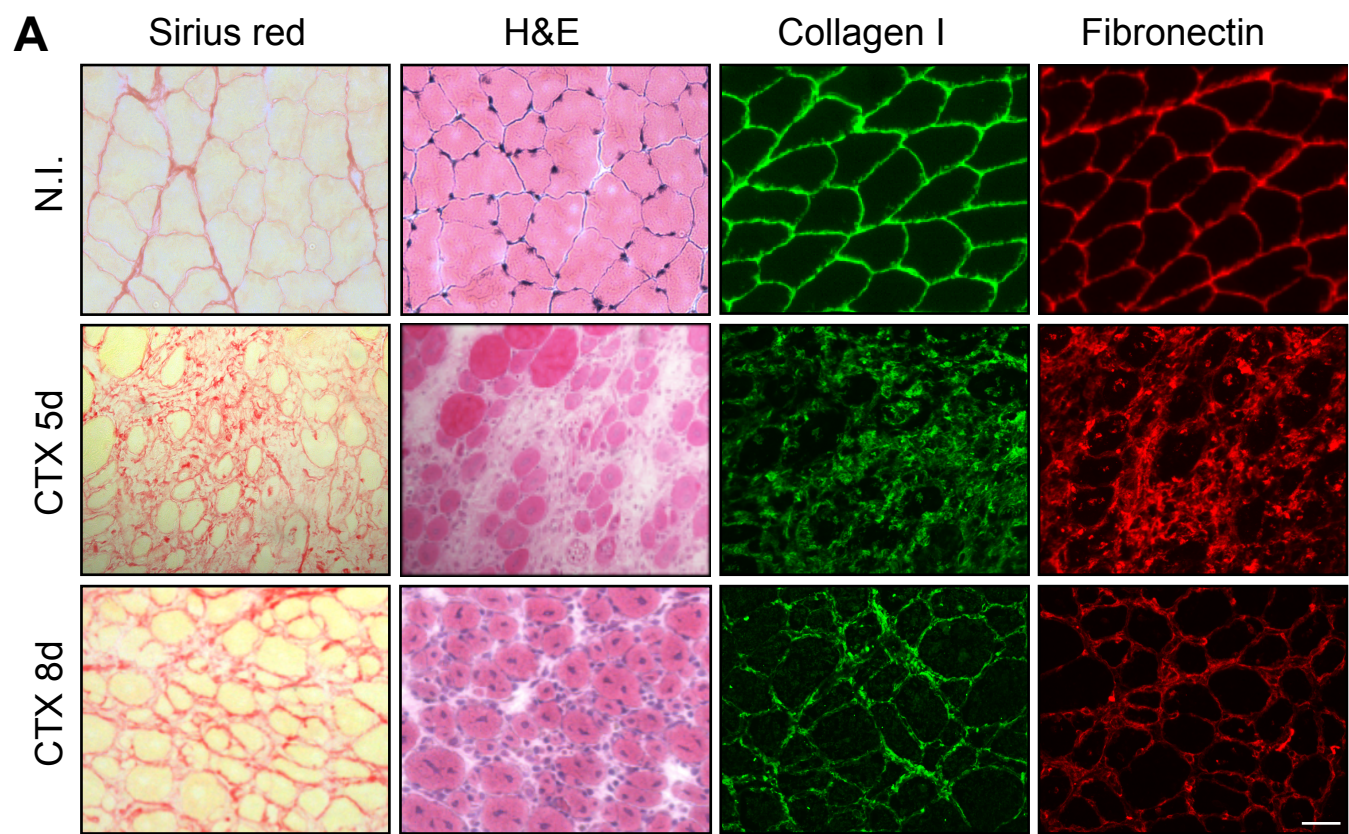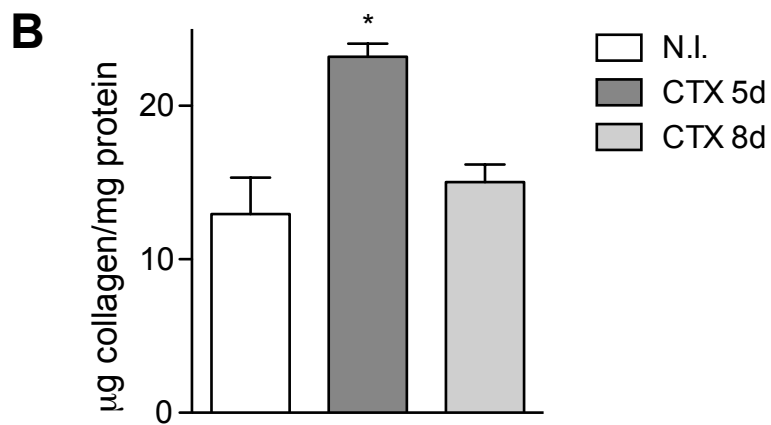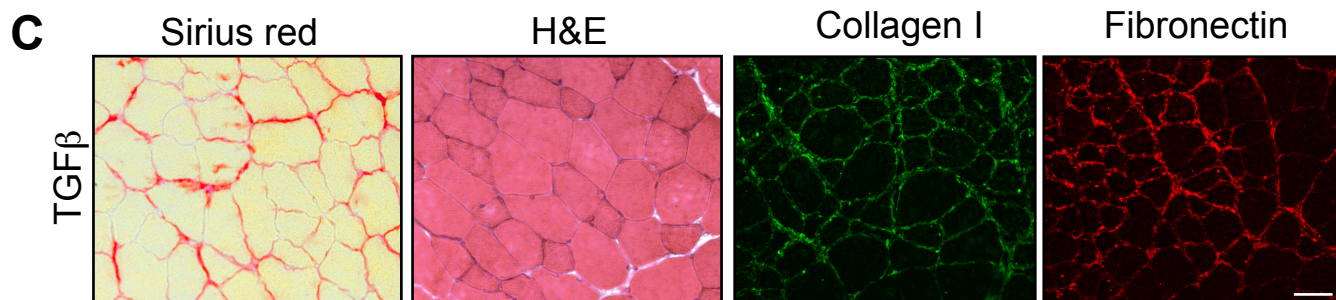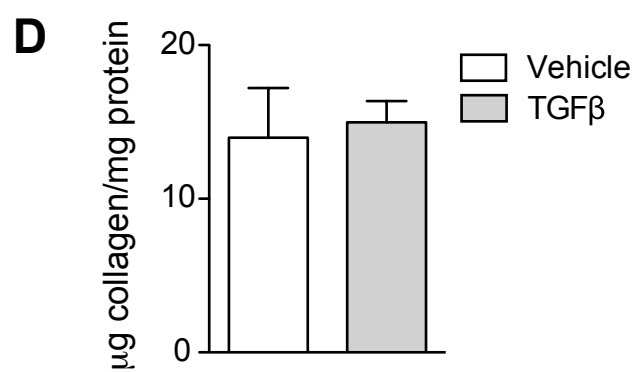

Supplement: Additional file 5: Figure S4 — Collagen deposition after cardiotoxin (CTX)-induced muscle injury and transforming growth factor beta 1 (TGFβ1) delivery alone is quickly resolved in wild-type (WT) mice. (A) Sirius red, hematoxylin and eosin (H&E), collagen I (green) and fibronectin (red) staining on WT tibialis anterior (TA) muscles after five and eight days from CTX injury, compared to non-injured (NI) muscle of sham-operated WT mice. (B) Quantification of collagen content in muscle after treatment. Data correspond to the mean ± SEM, n = 4 on each group. Non-parametric Mann–Whitney U test; *P <0.05 versus NI. (C) Sirius red, H&E, collagen I (green) and fibronectin (red) staining on WT TA muscle two weeks after two sequential treatments with recombinant TGFβ1 (50 ng in 50 μl phosphate-buffered saline (PBS)), spaced seven days apart. (D) Quantification of collagen content in muscle after injection of TGFβ1 or PBS (vehicle). Data are mean ± SEM, n = 4 for each group. Non-parametric Mann–Whitney U test; no significant differences P >0.05. Scale bars = 50 μm. [file 2044-5040-4-7-S5.pdf]

**A**

CTX

LAC

DEN

TGF $\beta$ 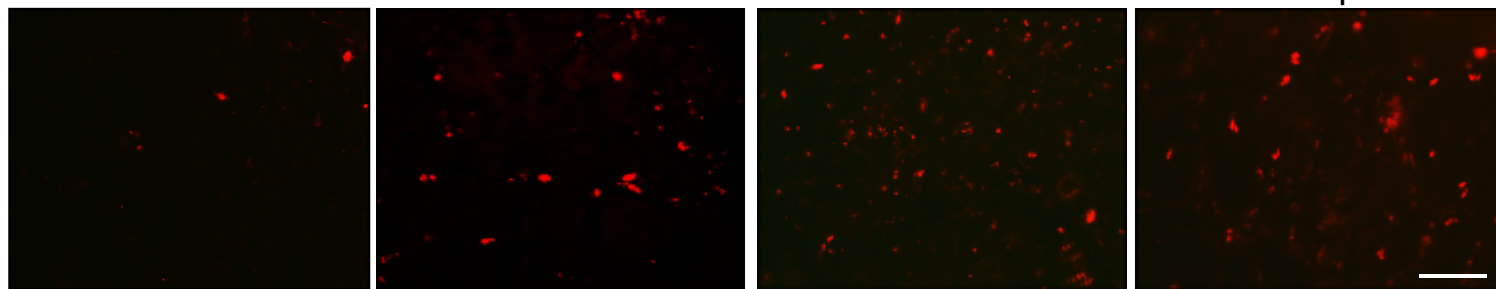

P-Smad2/3

**B**

CTX

LAC

CTX+DEN

CTX+TGF $\beta$ 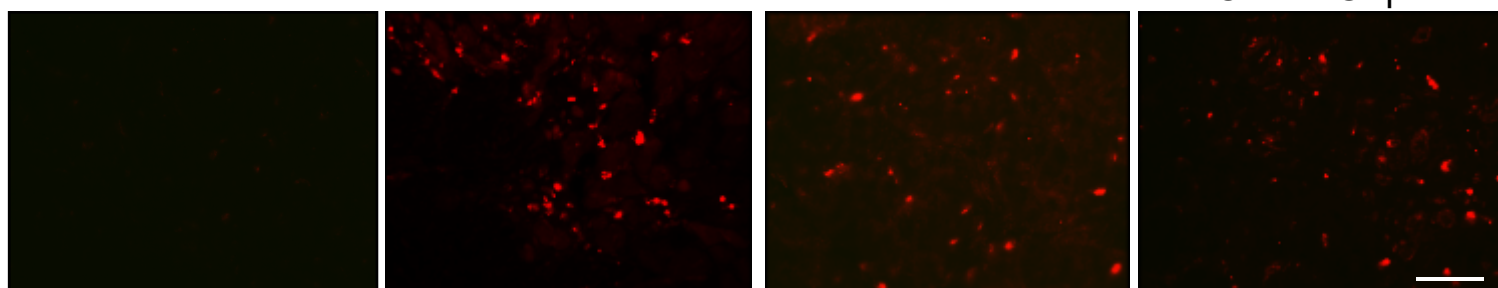

P-Smad2/3

Supplement: Additional file 6: Figure S5 — Smad2/3 protein phosphorylation in injured muscles. Immunofluorescence for phosphorylated-Smad2/3 proteins on sections from tibialis anterior (TA) muscle of mdx (A) and wild-type (WT) (B) mice after the indicated treatments. Scale bars = 50 μm. [file 2044-5040-4-7-S6.pdf]
